# *Erysipelothrix rhusiopathiae*-specific T-cell responses after experimental infection of chickens selectively bred for high and low serum levels of mannose-binding lectin

**DOI:** 10.1186/s13567-022-01126-w

**Published:** 2022-12-12

**Authors:** Eva Wattrang, Tina Sørensen Dalgaard, Rikke Brødsgaard Kjaerup, Mohammad Naghizadeh, Susanne Kabell, Helena Eriksson, Robert Söderlund

**Affiliations:** 1 Department of Microbiology, National Veterinary Institute, Uppsala, Sweden; 2 Department of Animal Science, Aarhus University, Tjele, Denmark; 3 Poultry Clinic, Blommenslyst, Odense, Denmark; 4 Department of Animal Health and Antimicrobial Strategies, National Veterinary Institute, Uppsala, Sweden; 5 Present Address: Centre for Medical Parasitology, University of Copenhagen, Copenhagen, Denmark

**Keywords:** Erysipelas, chicken, mannose-binding lectin, T-cell response

## Abstract

Erysipelas, caused by infection with *Erysipelothrix rhusiopathiae* (ER) is an important emerging disease in laying hens. We have earlier observed prominent mannose-binding lectin (MBL) acute phase responses in experimentally ER infected chickens. The present study aimed to further examine immune responses to ER by using chickens selectively bred for high (L10H) and low (L10L) serum MBL levels. Chickens were infected with ER at 3 weeks of age and immune parameters and bacterial load were monitored in blood until day 18 after infection. Blood and spleen leukocytes collected on day 18 were stimulated in vitro with ER antigens and blast transformation of different T-cell populations was assessed. The ER infection gave a very varied outcome and no clear differences were observed between L10H and L10L chickens with respect to leukocyte counts, bacterial load or clinical outcome. Nonetheless, rapid innate responses, e.g., heterophilia and increased serum MBL levels were noted in bacteraemic chickens. All ER infected chickens also showed transient increased expression of mannose receptor MRC1L-B and decreased expression of major histocompatibility complex II on monocytes day 1 after infection indicating monocyte activation or relocation. In vitro ER stimulation showed antigen specific blast transformation of CD4+, TCRγ/δ−CD8αβ+ and TCRγ/δ+CD8αβ+ spleen cells from all infected chickens. For CD4+ and TCRγ/δ−CD8αβ+ cells the proportions of blast transformed cells were significantly higher for samples from L10L chickens than those for samples from L10H chickens. This is the first observation of ER-specific T-cells in chickens and interestingly a Th1-type response comprising cytotoxic T-cells was indicated.

**Supplementary Information:**

The online version contains supplementary material available at 10.1186/s13567-022-01126-w.

## Introduction

Outbreaks of erysipelas, a disease caused by infection with the bacterium *Erysipelothrix rhusiopathiae* (ER), have been identified as a problem in laying hens in several European countries [1–7]. The perceived emergence/re-emergence of this disease in modern egg production has been associated with the transition from cage to floor housing and an increased risk of outbreaks has been identified for flocks with access to outdoor ranges [1–7]. In laying hen flocks erysipelas often manifests as rapidly progressing outbreaks with reduced egg production and high mortality often without clear clinical signs [1, 3, 4, 8]. Affected hens have an acute septicaemia and some birds may display unspecific clinical signs such as decreased appetite, depression, weakness, ruffled feathers and drooping tail and wings prior to death. Diagnosis is made on post-mortem findings indicative of septicaemia such as splenomegaly, hepatomegaly and petechial haemorrhages in internal organs combined with isolation of ER. The causative agent ER is a Gram-positive facultative anaerobic rod that can infect many different species with or without causing clinical disease [9]. The source and route of ER infection are in most cases unknown and although ER may survive in the environment for some time asymptomatic carriers of a variety of species also provide an important reservoir for the bacterium [9].

There is a long tradition of immunisation against erysipelas using both live and inactivated vaccines [10], mainly in pigs and turkeys. However, our understanding of host immune responses to ER infection and the development of protective immunity is still very limited. In chickens, a few studies have demonstrated induction of ER-specific antibody responses from approximately 1 week after experimental ER infection [11–13]. In addition, rapid and transient innate immune responses in the form of heterophilia, signs of monocyte activation, and increased blood concentrations of the acute phase protein mannose-binding lectin (MBL) were observed with onset 1 day after experimental ER infection of naïve chickens [13]. Such responses are expected upon infection with a bacterial pathogen where heterophils are considered important in the first line of defence as sentinel cells, regulators of immune responses and as effector cells by phagocytosing and killing infectious agents [14, 15]. Indeed, phagocytosing and killing of ER by neutrophils, the mammalian counterpart to chicken heterophils, has been shown by murine and porcine cells in vitro [16]. Moreover, in the naïve host innate opsonins such as complement and MBL can be important for effective phagocytosis of infectious agents [17, 18]. Early studies in mice indicated a role for complement in the innate response to ER infection in mice [19]. The C-type collectin MBL is a soluble pattern-recognition receptor with high affinity to carbohydrate residues on the surface of microorganisms and has direct opsonic properties as well as other roles in the innate elimination of pathogens [17, 20]. So far, chicken MBL has been found to have largely similar functions as mammalian MBL [20]. Interestingly, in previous experiments the relative increase in serum MBL concentration elicited by ER infection of chickens [13] was the most prominent infection induced increase we have monitored in this species so far, approximately threefold higher than that observed after *Escherichia coli* infection [21] and twofold higher than that typically observed after infectious bronchitis virus (IBV) infection [22–24]. We also found a strong positive correlation between serum MBL levels and the amount of ER detected in blood during the acute bacteraemia after ER infection [13]. Furthermore, the polysaccharide capsule of ER has a high content of mannose [25], which is a ligand for MBL [17, 20]. Thus, it could be fair to assume that MBL has a role in the chicken innate defence against ER infection by opsonisation for phagocytosis and one may hypothesise that high serum MBL levels would be an advantage for rapid clearance of the bacterium.

It has been shown in e.g., humans [26, 27], pigs [28, 29] and chickens [30, 31] that baseline MBL concentrations in serum are under genetic influence and show a large variation. In humans both the “high MBL” as well as the “low MBL” profile has been associated with seemingly pathogen-specific resistance against infections [26, 27]. In chickens the “low MBL” profile has been associated with increased susceptibility to *Salmonella enterica*, *Pasteurella multocida*, *E. coli* [20] and IBV infections [23, 24, 32]. Moreover, by selective breeding for high (H) and low (L) serum MBL concentrations two distinct chicken lines, L10H and L10L, have been created [30, 32] that have been used in studies of chicken immune responses to e.g. experimental infections and vaccination [23, 24, 30, 32].

To gain more insight into chicken immune responses to ER we therefore wanted to take advantage of the opportunity to use chickens with genetically determined high and low serum MBL level for experimental infection. The aim of the present study was to monitor both innate responses and subsequent development of ER specific immunity. For that purpose, chickens from both the L10H and L10L line were experimentally infected with ER and monitored for 18 days including frequent blood sample collection during the first week after infection. We have previously successfully assessed immune responses in ER infected chickens using an ER infection protocol where chickens were inoculated by intramuscular injection [13] and this model was therefore also chosen for the present study.

## Materials and methods

### Chickens and experimental design

The experimental chickens were offspring of the AU line L10 that has been selected for low (L) and high (H) serum levels of MBL for several generations [30, 32]. For this experiment 28 L10H and 23 L10L chicks, respectively, of generation 20 were hatched and subsequently raised under specific pathogen free conditions in a biocontained facility, group housed in pens on wood shavings with ad libitum access to feed and water on a diet that met or exceeded the Danish National Research Council requirements. At 3 weeks of age, experimental day −7, all chickens were weighed and chickens from each sub-line were divided into two groups to achieve an even weight distribution (Table 1). The numbers of chickens used was decided by power calculations (power 80%, sig. level 0.05, calculated with the R pwr package) based on earlier observed variations in heterophil and MBL-levels [13]. These calculations indicated that a minimum of 5 chickens per group was needed to observe statistically significant effects. Therefore, we aimed at minimum 6 individuals per sampling group and the numbers of chickens produced from the hatch decided the final number of chickens used.

**Table 1:** **Experimental outline with procedures undertaken in the experimental groups**

Group	Sampling group	Sex^a^	MHC^b^	Experimental day																			
				− 7	0	1	2	3	4	5	6	7	8	9	10	11	12	13	14	15	16	17	18
L10H inf.	**A** *n = 7*	F 5, M 2	193/193 5, 193/448 2	W	BI	B		B							BW				B				BWP
	**B** *n = 7*	F 3, M 4	193/193 5, 193/448 2	W	BI		B		B			B			BW				B				BWP
L10L inf.	**A** *n = 6*	F 4, M 2	193/193 6	W	BI	B		B							BW				B				BWP
	**B** *n = 6*	F 1, M 5	193/193 6	W	BI		B		B			B			BW				B				BWP
L10H uninf.	**A** *n = 7*	F 3, M 4	193/193 4, 193/448 3	W	B	B		B							BW				B				BWP
	**B** *n = 7*	F 3, M 4	193/193 3, 193/448 4	W	B		B		B			B			BW				B				BWP
L10L uninf.	**A** *n = 6*	F 4, M 2	193/193 6	W	B	B		B							BW				B				BWP
	**B** *n = 5*	F 3, M 2	193/193 5	W	B		B		B			B			BW				B				BWP

L10H infected (inf.), L10L infected, L10H uninfected (uninf.) and L10L uninfected with sampling groups A and B, respectively, on the indicated experimental days.

^a^Number of chickens of each sex in the respective sampling groups, F = female, M = male. Sex was determined at post-mortem examination.

^b^Number of chickens of each MHC haplotype in the respective sampling groups, 193/193 = 193 bp homozygous, 193/448 = 193 bp/448 bp heterozygous. For details see “Materials and methods”.

*W* weighing, *B* blood sampling, *I* infection, *P* post-mortem examination and spleen collection.

On experimental day 0 one group of each sub-line (i.e., L10L and L10H) were infected by intramuscular injection of 1.5 × 10^8^ colony forming units (cfu)/mL ER in 0.5 mL broth and the other groups were kept as uninfected controls and injected with 0.5 mL sterile broth. Infected and uninfected chickens were housed separately in two sections of the biosecure facility.

Blood samples were collected on experimental days 0, 1, 2, 3, 4, 7, 10, 14 and 18. Blood samples were drawn by needle and syringe under sterile conditions from the jugular vein and approx. 500 µL blood/sampling were collected from each chicken. The blood was divided into sterile blood collection tubes with 1.0 mg EDTA K_2_ as additive and into sterile test tubes without additives, respectively. In addition, on experimental day 18 samples were divided 3-ways and sterile blood collection tubes with 37 USP units of lithium heparin were used for the third part. During the intense sampling period experimental day 1–4 blood samples were collected from alternate sampling groups of 7–5 chickens in each group in order to minimise the impact of the procedure on the chickens (Table 1). At day 7 four randomly selected L10H chickens were sacrificed by cervical dislocation, two from the infected sampling group B and two from the uninfected sampling group B (Table 1). All remaining chickens were killed by cervical dislocation on day 18 after infection. Macroscopic post-mortem examinations were performed by a poultry pathologist according to the in-house protocol at the Poultry Clinic (Odense, Denmark) on all chickens that died/were sacrificed during the experiment and at the end of the experiment.

Chickens were observed for clinical signs of disease two or three times daily on experimental days 1–7 and once daily during the rest of the experiment and weighed on experimental days −7, 10 and 18.

### Culture of ER inoculate and re-isolation of ER in samples from infected chickens

The ER strain 15-ALD003475, derived from an outbreak of erysipelas in a Swedish laying hen flock in 2015 and previously evaluated in experimental infection of chickens [13, 33] was used in the present experiment. This strain was determined to be of an “intermediate” lineage (unpublished data) according to whole-genome single-nucleotide polymorphism comparison with isolates from the study by Forde et al. [34]. For the inoculate, bacteria from a 24 h culture on sheep blood agar were cultured for 24 h in 0.1% Tween 80, 0.1% d-glucose and 20 mg/L l-tryptophane supplemented tryptic soy broth (#B321730, National Veterinary Institute, Uppsala, Sweden) as previously described [13]. A 0.5 mL volume of inoculate was used to infect each chicken intra-muscularly in the breast muscle directly after the 24 h culture and numbers of ER in the inoculate was subsequently determined by serial dilution and culture as previously described [13].

Growth of ER colonies was quantified in EDTA-stabilised blood samples by direct culture and ER DNA was detected and quantified by PCR methodology as previously described [33]. Liver and spleen samples for ER-culture and/or PCR-analysis were collected at post-mortem examination. From chickens that died or were euthanised on experimental days 3 to 7, samples for direct culture of liver were collected and liver samples were also collected into selective sodium-azide crystal-violet broth (#B321051/5, National Veterinary Institute; containing 5 µg/mL crystal-violet and 0.2 mg/mL sodium-azide) and cultured as previously described [13]. Liver and spleen samples were also collected and frozen at −70 °C for PCR analysis. From chickens killed on experimental day 18, only liver samples for culture in selective broth and spleen and liver samples for PCR analysis were collected. For PCR analysis of organ samples, DNA was isolated using the IndiMag Pathogen kit (Indical) on a Maelstrom-9600 automated system and quantitative real-time ER PCR [33] was performed on all samples. Organ samples positive for ER DNA in real-time PCR were subsequently analysed using digital droplet PCR (ddPCR) for quantification of ER DNA [33] and chicken glyceraldehyde 3-phosphate dehydrogenase (GAPDH) DNA [35] and the amount of ER DNA in the tissue was expressed as the ratio of ER copies/chicken GAPDH copies as previously described for quantification of *Eimeria tenella* DNA in chicken tissues [35].

Suspected ER colonies detected by culture methods were verified by matrix-assisted laser desorption/ionization–time-of-flight mass spectrometry (MALDI–TOF MS) on a Biotyper instrument (Bruker).

### Blood leukocyte counts

Absolute counts of heterophilic granulocytes, monocytes and thrombocytes in EDTA-stabilised whole blood samples were determined using a previously described no-lyse, no-wash flow cytometry based method [13]. Two panels of monoclonal antibodies were used in this analysis (Table 2) and in addition to the aforementioned leukocyte populations also sub populations of lymphocytes, i.e. B-cells, TCRγ/δ+, CD4+, CD4−CD8αβ+ (cytotoxic T lymphocytes; CTL) and CD4−CD8αα+ cells, were identified and CD25 expression on TCRγ/δ+, CD4+, CD4−CD8αβ+ and CD4−CD8αα+ cells and major histocompatibility complex (MHC) II expression on B-cells and monocytes were analysed as described in the gating strategies in Additional files 1 and 2. Samples were recorded for 1 min at reduced flow rate (corresponding to approximately 20 000 events in the CD45+ gate in panel 1 and approximately 10 000 events in the lymphocyte gate in panel 2, respectively, Additional files 1 and 2) in a BD FACSCanto™ (BD Biosciences), equipped with 488 nm blue and 633 nm red lasers and results were analysed using the FACSDiva (BD Biosciences) software. The number of events counted in the bead gate (Additional files 1 and 2) was used to determine the volume of blood sample analysed and calculate absolute numbers of the leukocyte populations. The fluorescent beads used were 123 count eBeads (#01-1234-42, Invitrogen, Thermo Scientific). Single-stained compensation controls and fluorescence minus one (FMO) negative controls were included in the assays. Titrations of all antibodies were performed to determine optimal labelling conditions prior to the experiment. Calibration beads (BD™ CS&T RUO beads #661414, BD Biosciences) were used to check the cytometer performance and make adjustments, ensuring consistent values from day to day.

**Table 2:** **Monoclonal antibodies used for immunolabelling and combinations (panels) used to phenotype leukocytes**

Abbreviation	Clone	Specificity	Fluorochrome	Panel		
				1	2	3
CD41/61−Fitc	11C3^a^	Chicken CD41/61 integrin (GPIIb-IIIa)	Fluorescein^c^	X	–	–
KUL01-RPE	KUL01^b^	Chicken mannose receptor MRC1L-B [36]	R-phycoerythrin^c^	X	–	–
MHCII−PerCp/Cy5.5	2G11^b^	Chicken MHC class II	Peridinin chlorophyll-cyanine 5.5^d^	X	–	–
CD45−PE/Cy7	UM16-6^a^	Chicken CD45, all isoforms [37]	R-phycoerythrin-cyanine 7^d^	X	–	–
Bu1-APC/Cy7	AV20^b^	Chicken Bu-1 (chB6) transmembrane protein	Allophycocyanin-cyanine 7^d^	X	–	–
CD4−Fitc	CT-4^b^	Chicken CD4	Fluorescein^c^	–	X	–
CD8−RPE	EP42^b^	β-chain of chicken CD8	R-phycoerythrin^c^	–	X	X
TCRγ/δ−PerCp/Cy5.5	TCR-1^b^	Chicken γ/δ T-cell receptor	Peridinin chlorophyll-cyanine 5.5^d^	–	X	–
CD8α−Cy5	3-298^b^	α-chain of chicken CD8	Cyanine 5^c^	–	X	X
CD25−PE/Cy7	AV142^a^	Chicken CD25, interleukin-2 receptor α-chain	R-phycoerythrin-cyanine 7^c^	–	X	–
TCRγ/δ−Fitc	TCR-1^b^	Chicken γ/δ T-cell receptor	Fluorescein^c^	–	–	X
CD4−PACBLU	CT-4^b^	Chicken CD4	Pacific Blue™^c^	–	–	X

Panels 1 and 2 were used for whole blood samples, panel 3 was used for activated T-cells in culture.

X: used in panel; –: not used in panel.

^a^Purchased from Bio-Rad Antibodies.

^b^Purchased from SouthernBiotech.

^c^Fluorochrome conjugated by manufacturer.

^d^Fluorochrome conjugated using Lightning-Link® conjugation kits (abcam) according to the manufacturer’s protocol.

### Leukocyte isolation and assessment of in vitro T-cell activation with ER antigen

At day 18 after infection spleens and heparinised blood samples were collected sterilely from all remaining chickens. Spleens were collected into test tubes with sterile phosphate buffered saline (PBS; without Ca^2+^ and Mg^2+^ at pH 7) and stored on ice until processing. After removal of a spleen sample, approximately 1/10 of the spleen tissue, for PCR analysis the remaining spleen tissue was used to prepare a single spleen cell suspension and mononuclear cells were isolated by Ficoll-Paque PLUS (GE Healthcare Life Sciences) gradient centrifugation as previously described [38]. Heparinised blood was diluted 1:1 in PBS and peripheral blood mononuclear cells (PBMC) were isolated by Ficoll-Paque PLUS gradient centrifugation according to the same procedure as the spleen mononuclear cells.

For both spleen cells and PBMC, cells were suspended in growth medium, i.e. RPMI 1640 (BioWhittaker®, Lonza) supplemented with 200 IU penicillin/mL, 100 µg streptomycin/mL and 5% heat inactivated foetal calf serum (FCS; BioWittaker® #DE14-801 F, Lonza) and the final mononuclear cell concentration was adjusted to 10^7^ live cells/mL. One hundred µL of cell suspension and 100 µL of growth medium alone, or supplemented with sonicated ER antigen at a final concentration of 0.5 µg/mL were added per well in round-bottomed microtiter plates (Nunc™, ThermoFisher Scinetific). The ER antigen was prepared from bacterial colonies suspended in buffer, sonicated and subsequently centrifuged to remove particulate matter according to an earlier described protocol [13]. Cultures were incubated for 72 h at 40 °C, 5% CO_2_ in a humid atmosphere, and cells were subsequently analysed by flow cytometry.

Immunofluorescence labelling was performed according to a protocol described earlier [39]. In brief, cultured mononuclear cells were incubated with antibody panel 3 (Table 2) to identify CD4+, TCRγ/δ−CD8αβ+ (CTL), TCRγ/δ−CD8αα+, TCRγ/δ+CD8αβ+, TCRγ/δ+CD8αα+ and TCRγ/δ+CD8− cells combined with LIVE/DEAD® fixable Aqua dead stain (#L34957, ThermoFisher Scientific) for dead cell exclusion for 20 min at room temperature in the dark. Cells were subsequently washed and analysed directly.

Flow cytometry was performed using a BD FACSCelesta™ Cell Analyzer (BD Biosciences), equipped with 488 nm blue, 633 nm red and 405 nm violet lasers and results were analysed using the FACSDiva (BD Biosciences) software. Single-stained compensation controls and fluorescence minus one (FMO) negative controls were included in the assays, the gating strategies are shown in Additional file 3 and approximately 100 000 events for PBMC samples and 50 000 events for spleen samples, respectively, were recorded in the live gate. Titrations of all antibodies were performed to determine optimal labelling conditions prior to the experiment. Calibration beads were used to check the cytometer performance as described above.

### ELISAs for detection of chicken MBL, IgY and IgM antibodies to ER

The MBL serum concentration was measured using an earlier described in house ELISA based on the anti-chicken cMBL antibody HYB182-01 from BioPorto A/S [22, 40].

An earlier described in house ELISA methodology for detection and quantification of IgY antibodies to ER in chicken serum was used [13, 41]. In brief, the method uses a sonicated ER preparation, in the present study derived from the challenge strain 15-ALD003475, as coating antigen and horseradish peroxidase conjugated polyclonal goat anti chicken IgG (IgY)-Fc antibodies (#AAI29P, BioRad Antibodies) as tracer antibody. Serum samples are titrated in twofold steps starting at dilutions 1:100 or 1:1000 depending on antibody concentration, to achieve a dilution curve. For each sample absorbance values were plotted against the sample dilution and the equation for the linear part of the curve was determined by regression analysis. Antibody titers were then calculated as the dilution that would achieve an absorbance value of 1. For detection and quantification of IgM antibodies to ER, horseradish peroxidase conjugated polyclonal goat anti chicken IgM antibodies (#AAI27P, BioRad Antibodies) was used as tracer antibody in the above protocol.

### *MHC-B* genotyping

Putative MHC genotypes of L10 chickens have previously been shown to comprise 194 bp (also called BW3-like) and 448 bp (also called B6-like) haplotypes [24] using LEI0258 microsatellite locus [42] genotyping. The individual MHC genotypes of chickens in the present experiment were likewise determined by PCR-based fragment analysis [43] as previously described [44].

### Data analysis

Numerical data were analysed as mean values ± 95% confidence intervals (CI) and mean values with non-overlapping CI were treated as rejecting the null hypothesis of no difference. For antibody titers geometrical mean values were calculated, for all other data arithmetic mean values were used. Geometric mean values and CI for geometric mean values were calculated using the software package R 3.5.0. Results from ER induced blast transformation were calculated as net proportions (i.e. the proportion of blasts in stimulated cultures minus the proportion of blasts in medium cultures). Since the CI of these net proportions were asymmetrical, and thus non-normally distributed, the arithmetic means and CI were estimated based on square-root transformation, using a Microsoft Excel spreadsheet that implements the method of Land [45]. Pearson correlation was calculated using the software package R 3.5.0. A generalized linear model was fit to categorical data with the glm function in R 4.0.4 with disease status, i.e. chickens with clinical signs and/or bacteraemia versus chickens with no clinical signs or bacteraemia, as the binary outcome and chicken subline, i.e. L10H or L10L, and sex as categorical predictors. The interaction between sex and lineage was deemed biologically plausible and included in the analysis.

## Results

### Clinical outcome of the infection, re-isolation of ER and post-mortem findings

Chickens were observed for clinical signs of disease throughout the experiment and clinical signs, post-mortem findings for all chickens that tested positive for ER by culture at one or more occasions during the experiment are listed in Additional file 4. No clinical signs were observed in any of the chickens on day 1 or 2 after infection. On day 3 one chicken in the L10H group was found dead in the morning and three chickens, one L10H and two L10L showed moderate clinical signs and either died immediately after blood sampling (L10L) or died/was euthanized later during day 3. On day 4 three chickens (two L10H and one L10L) showed clinical signs and one L10L chicken died immediately after blood sampling without showing clinical signs of disease. On days 5 and 6 three L10H chickens showed clinical signs. On day 7 one L10H chicken showed clinical signs and from day 8 onwards no clinical signs were observed for any of the chickens. None of the uninfected chickens showed any clinical signs of disease.

Chickens were weighed on experimental days −7, 10 and 18 and neither group mean values of body weight nor daily weight gain differed between ER-infected and uninfected chickens throughout the experiment (Additional file 5). Nonetheless, two of the infected L10H chickens that showed clinical signs of disease for 3 to 4 days, had pronounced lower daily weight gain between day −7 and day 10 of the experiment (Additional file 5C).

**Figure 1:**
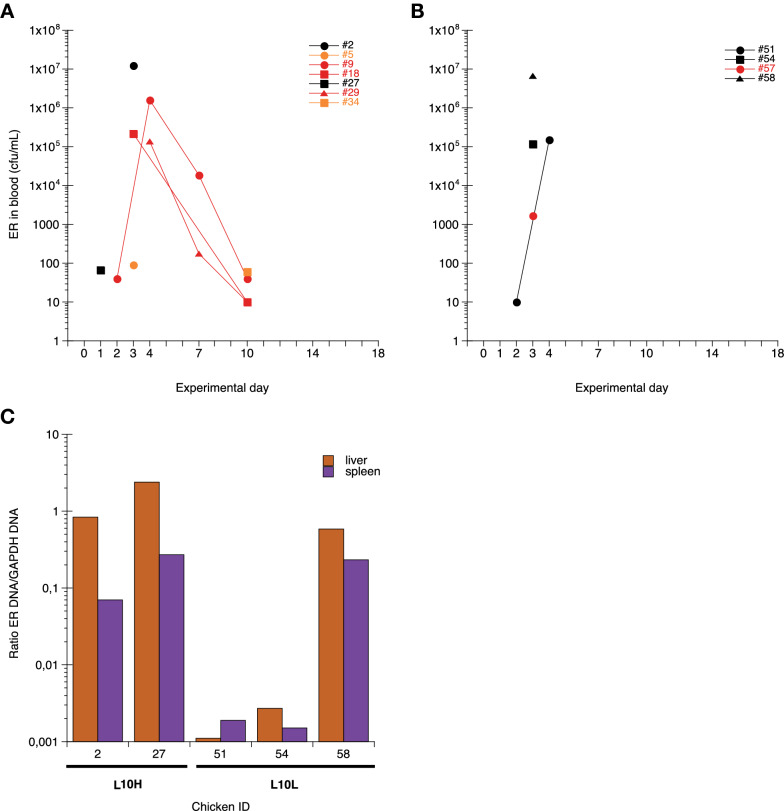
**Detection of ER in blood (A, B) and in spleen and liver tissues (C).** **A**, **B** ER (cfu/mL) detected by direct culture of blood collected on the indicated days after ER infection on day 0. Values for individual L10H (**A**) and L10L (**B**) chickens are shown with black symbols for chickens that died, red symbols for chickens with clear clinical signs of disease at one or more occasions during the experiment and orange symbols for chickens without clear clinical signs of disease. **C** Relative amounts of ER DNA (ratio of ER DNA/chicken GAPDH DNA) quantified by ddPCR in livers (brown bars) or spleens (purple bars) collected from the indicated individual chickens that were found dead on day 3 (#2 and 27), euthanized on day 3 (#58) or died at blood sampling on day 3 (#54) or day 4 (#51) after ER infection on day 0.

By direct culture of blood, ER was isolated from in total eleven, seven L10H and four L10L, individual ER infected chickens on one or more occasions during the experimental period (Figures 1A and B). None of the uninfected chickens were positive for ER in blood at any time point. The highest numbers of ER in blood were detected on days 3–4 after infection in both groups and the numbers of bacteria in blood correlated with the severity of clinical signs (Figures 1A and B, Additional file 4). Liver samples from the chickens that were found dead, euthanized due to severe clinical signs or those that died at blood sampling on days 3 and 4 after infection were positive for ER by culture and for ER DNA by PCR (Additional file 4). Using ddPCR for ER and chicken GAPDH the amount of ER DNA was quantified in liver and spleen samples (Figure 1C). Results showed that the relative amounts of ER DNA were between 37- and 2000-fold higher in organs from chickens found dead or euthanized due to severe clinical signs compared to those that died at blood sampling. On day 7 after infection four random L10H chickens, two infected and two uninfected, were sacrificed as planned and liver and spleen samples were tested for ER by culture and/or by PCR and all of these chickens were negative for ER. Likewise on day 18, all remaining chickens were tested for ER in liver and spleen samples by culture and/or PCR and all were negative for ER.

Post-mortem examinations were performed on all chickens in this experiment. The three chickens that died on day 3 after infection all showed pathological lesions consistent with septicaemia, e.g. enlarged spleen and liver. Both chickens that died after blood sampling had blood clots on the liver surface (Additional file 4). Hence, it seems that the two latter chickens died of liver rupture rather than septicaemia, which is also supported by the lower ER DNA load in liver and spleen (Figure 1C).

None of the four random chickens sacrificed on day 7 or the chickens sacrificed on day 18 had any pathological lesions consistent with ER infection.

Thus, the majority of ER infected chickens in both groups showed no clinical signs (64% of L10H and 67% of L10L) and at least 50% did not have bacteraemia (50% of L10H and 67% of L10L) at any of the sampling occasions. Both groups had chickens that died/were euthanized due to septicaemia (14% L10H and 8% L10L) and chickens that showed reversible clinical signs of disease (21% L10H and 8% L10L) but only the L10H group comprised chickens with reversible clinical signs that lasted more than 1 day (14%) and chickens with bacteraemia without clear clinical signs (14%). In addition, the L10L group had two chickens (12%) with bacteraemia that died from liver rupture at blood sampling. Overall, infected L10L chickens had a lower proportion of chickens with clinical signs and/or bacteraemia at one or more occasions during the experiment, 33%, compared to infected L10H chickens, 50%, but this difference was not statistically significant (GLM estimate −1.504, *p* = 0.219). Moreover, statistically significantly lower proportions of male chickens, 23%, had clinical signs and/or bacteraemia at one or more occasions during the experiment compared to female chickens, 67% (GLM estimate −2.708, *p* = 0.0475). An interaction between chicken subline and sex was also observed with more male L10L chickens with clinical signs and/or bacteraemia compared to male L10H chickens (GLM estimate 2.197, *p* = 0.233) but this was not statistically significant.

### Blood leukocyte counts during ER infection

**Figure 2:**
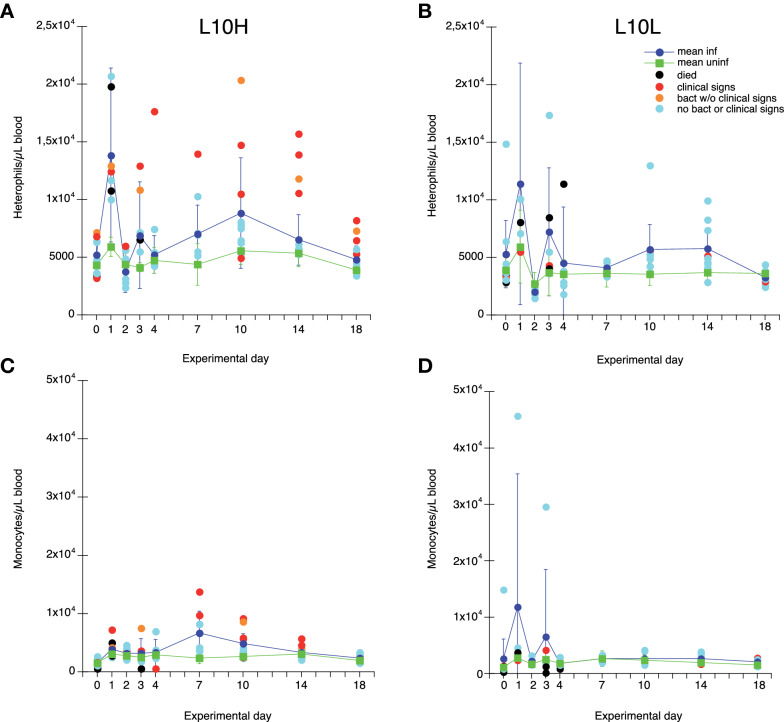
**Numbers of heterophils (A, B) and monocytes (C, D) in blood.** Results from L10H (**A**, **C**) and L10L (**B**, **D**) chickens at the indicated days after ER infection on day 0. Results are mean values ± 95% CI for infected chickens (dark blue circles) and uninfected chickens (green squares), where non-overlapping CI indicate statistically significant differences, and individual values for infected chickens. Black circles: chickens that eventually died, red circles: chickens that showed clear clinical signs of disease at one or more occasions, orange circles: chickens positive for ER in blood at one or more occasions without clear clinical signs of disease, light blue circles: infected chickens without clinical signs of disease or bacteraemia. Monoclonal antibody panels for immunolabelling are described in Table 2 and gating strategies in Additional files 1 and 2.

Absolute counts of circulating heterophils, monocytes, thrombocytes and different lymphocyte sub populations (flow cytometry gating in Additional files 1 and 2) were analysed in blood samples collected on days 0, 1, 2, 3, 4, 7, 10, 14 and 18. For blood heterophils, a general transient increase in cell numbers was observed for both L10H and L10L infected chickens on day 1 after ER infection but this was not statistically significantly different from heterophil numbers of uninfected chickens (Figures 2A and B). Chickens that experienced clinical signs and/or bacteraemia tended to have high heterophil numbers, which was particularly evident for the L10H group. Blood monocyte numbers were not statistically significantly different between infected and uninfected chickens (Figures 2C and D). However, L10H chickens with clinical signs and/or bacteraemia tended to have higher monocyte numbers with a peak on day 7 after ER infection. Blood thrombocyte numbers varied for both infected and uninfected chickens and did not seem influenced by the ER infection (Additional file 6). Even so, a L10H chicken that died on day 3 showed clear thrombocytopenia on day 3 and one infected L10L chicken without symptoms and a L10L chicken that was euthanized on day 3 also showed low thrombocyte numbers on days 2 and 3, respectively.

For lymphocyte subpopulations (Additional file 7), the majority showed no statistically significant differences between ER-infected and uninfected chickens of either group. Exceptions from this were observed for CD4−CD8αβ+ cells, i.e. CTL, that were significantly higher in blood from infected L10H chickens compared to the uninfected controls on day 10 after infection (Additional file 7G). Moreover, for many of the subpopulations some of the individual ER-infected chickens of both groups showed pronounced low numbers of cells on days 3–4 after infection. In general, most of the identified lymphocyte sub populations showed increasing numbers during the experimental period for both infected and uninfected chickens.

Thus, no clear pronounced changes in blood leukocyte counts were observed upon the ER-infection. However, trends for high heterophil and monocyte numbers as well as low lymphocyte numbers in bacteraemic chickens were noted.

### Expression of MRC1L-B and MHCII on circulating monocytes during ER infection

**Figure 3:**
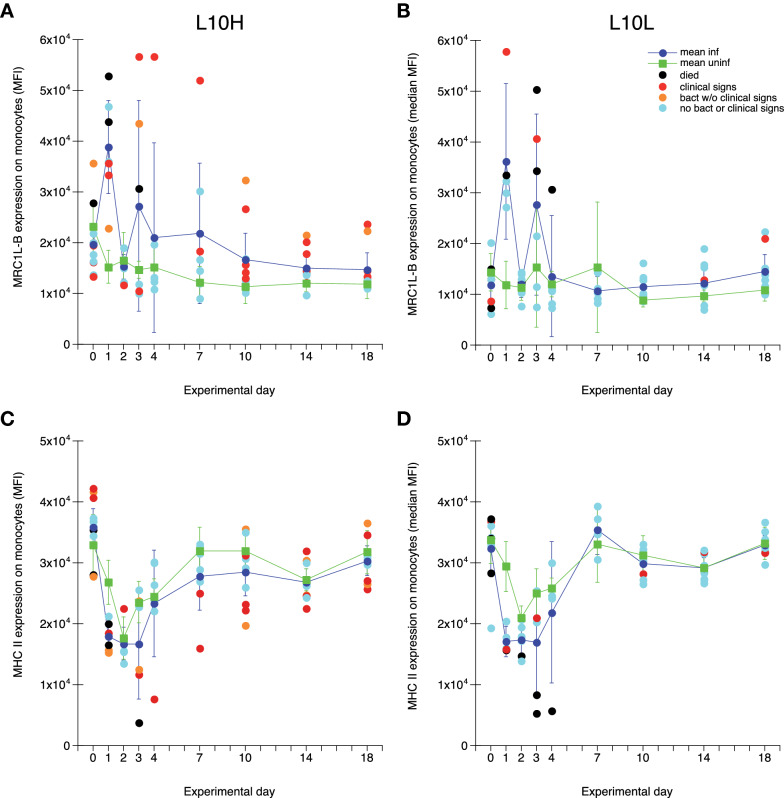
**Expression of MRC1L-B (A, B) and MHCII (C, D) on monocytes in blood.** Results from L10H (**A**, **C**) and L10L (**B**, **D**) chickens at the indicated days after ER infection on day 0. Results are mean values ± 95% CI for infected chickens (dark blue circles) and uninfected chickens (green squares), where non-overlapping CI indicate statistically significant differences, and individual values for infected chickens. Black circles: chickens that eventually died, red circles: chickens that showed clear clinical signs of disease at one or more occasions, orange circles: chickens positive for ER in blood at one or more occasions without clear clinical signs of disease, light blue circles: infected chickens without clinical signs of disease or bacteraemia. Monoclonal antibody panels for immunolabelling are described in Table 2 and gating strategies in Additional files 1 and 2.

Levels of cell surface expression of the mannose receptor MRC1L-B and MHCII on circulating monocytes detected by fluorochrome conjugated antibodies described in Table 2 was monitored as median fluorescence intensity (MFI) in the monocyte gate (Additional file 1). Results showed that the expression of MRC1L-B was statistically significantly increased on monocytes from both infected L10L and L10H chickens on day 1 after ER infection compared to that of uninfected chickens (Figures 3A and B). Several of the individual infected chickens that displayed clinical signs of disease and/or were bacteraemic during the experiment also showed high MRC1L-B expression on days 3–7 after infection. The MHCII expression on monocytes showed a large variation between days for all chickens (Figures 3C and D). Nevertheless, a significantly lower MHCII expression was observed for monocytes from infected chickens of both groups on day 1 after ER infection compared to that of uninfected chickens. Moreover, markedly low MHCII expression was observed on days 3–7 after ER infection on monocytes from chickens that displayed clinical sings of disease and/or were bacteraemic during the experiment. On cellular level including data from all sampling occasions, a statistically significant negative correlation between MRC1L-B and MHCII expression levels was observed for monocytes from infected chickens but not for monocytes from uninfected chickens: L10H infected *r *= −0.56 (−0.70 to −0.39; *p* < 0.001, Pearson correlation coefficient with 95% CI); L10L infected *r *= −0.56 (−0.71 to −0.36; *p* < 0.001); L10H uninfected *r* = 0.11 (−0.32 to 0.11; *p* = 0.32); L10L uninfected *r *= −0.19 (−0.41 to 0.06; *p* = 0.13). Hence, signs of activation and/or recruitment of different monocyte subpopulations were observed for the ER infected chickens.

### CD25 expression on circulating T-cell subpopulations during ER infection

The proportion of CD25 expressing cells (gating strategy in Additional file 2) was determined for CD4+CD8− cells, CD4−CD8αβ+ cells, i.e. CTL, and for total TCRγ/δ+ cells (Additional file 8). The number of events recorded for CD4−CD8αα+ cells was very low, around 8 events, and therefore it was not meaningful to analyse for CD25 expression on this population. The proportion of CD25+ cells were approximately 2%, 0.2%, and 2% for CD4+CD8− cells, CD4−CD8αβ+ cells and TCRγ/δ+ cells, respectively, in blood from uninfected chickens of both L10 subgroups. Pronounced increases in CD25+ cells were observed on days 7 and 10 after infection for some of the individual L10H chickens that displayed clinical sings of disease and/or were bacteraemic during the experiment (Additional file 8A, C and E). These increases were particularly high for TCRγ/δ+ cells where some of these individuals showed 20–70% CD25+ cells. In general, the proportions of CD25+ T-cells were not statistically significantly different between ER-infected and uninfected chickens except for CD4+CD8− cells that had statistically significantly higher proportions of CD25+ cells in blood from infected L10H chickens on day 10 compared to those from uninfected L10H chickens (Additional file 8A).

Hence, some evidence of activation in the form of CD25 expression on circulating T-cells were observed in chickens that displayed clinical signs and/or bacteraemia during the experiment.

### Systemic MBL responses during ER infection

**Figure 4:**
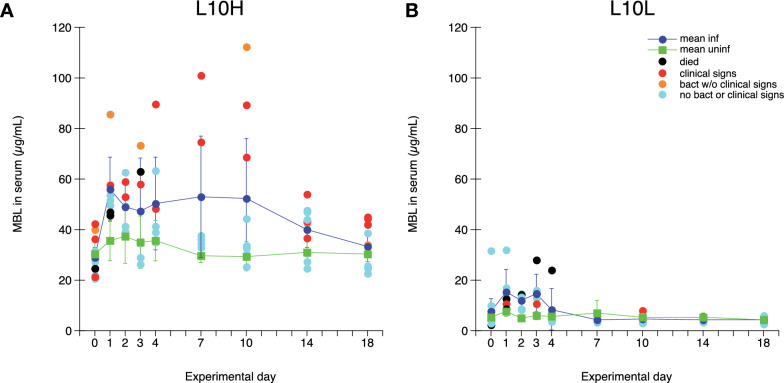
**Concentration of MBL (µg/mL) in serum.** Results from L10H (**A**) and L10L (**B**) chickens at the indicated days after ER infection on day 0. Results are mean values ± 95% CI for infected chickens (dark blue circles) and uninfected chickens (green squares), where non-overlapping CI indicate statistically significant differences, and individual values for infected chickens. Black circles: chickens that eventually died, red circles: chickens that showed clear clinical signs of disease at one or more occasions, orange circles: chickens positive for ER in blood at one or more occasions without clear clinical signs of disease, light blue circles: infected chickens without clinical signs of disease or bacteraemia.

The MBL concentration in serum was determined in blood samples collected during the experiment (Figure 4). As expected MBL levels for uninfected chickens were in average sixfold higher for L10H chickens at approximately 30 µg/mL compared to L10L chickens at approximately 5 µg/mL. At day 1 after ER infection the average serum MBL levels for infected chickens increased approximately twofold compared to uninfected chickens for both L10 subgroups. The post-infection MBL levels showed a large variation between individuals and were not statistically significantly different from those of uninfected chickens except for L10L chickens on day 2 after infection. For the ER infected L10L chickens average serum MBL levels remained increased until day 3 after infection whereafter they returned to pre-infection levels for the rest of the experimental period (Figure 4B). For the ER infected L10H chickens average serum MBL levels remained increased until day 10 after infection. Some of the chickens that displayed clinical sings of disease and/or were bacteraemic during the experiment showed MBL levels in the region of threefold higher than pre-infection during that time (Figure 4A).

Thus, the ER infection induced prompt MBL responses in both groups of chickens and individuals with a prolonged course of infection showed more pronounced and longer lasting responses.

### Identification of ER specific T-cells responding to in vitro antigen re-stimulation

Mononuclear cells isolated from spleens and blood at day 18 after ER infection were cultured in the presence or absence of ER antigens for 72 h. Six different lymphocyte sub populations, i.e. CD4+, TCRγ/δ−CD8αβ+ (CTL), TCRγ/δ−CD8αα+, TCRγ/δ+CD8−, TCRγ/δ+CD8αβ+, and TCRγ/δ+CD8αα+ cells were identified by immunofluorescence labelling using antibody panel 3 (Table 2) and assessed for blast transformation (high FSC and SSC) by flow cytometry (Additional file 3). The proportions of the different lymphocyte populations out of live cells in the spleen and PBMC cultures are shown in Additional file 9A and B. In spleen cell cultures (Additional file 9A), TCRγ/δ−CD8αβ+ cells comprised the largest population identified at approximately 40% followed by CD4+ cells at approximately 20% while the remaining four populations were markedly smaller at approximately 5% or less of the live cells in the cultures. Spleen cell cultures from both uninfected and infected L10H and L10L chickens, respectively, had very similar proportions of the different lymphocyte subpopulations when cultured in medium without ER antigen. For spleen cell cultures stimulated with ER antigen the proportions of TCRγ/δ+CD8αβ+ cells tended to be higher than those in medium cultures for all types of chickens but no statistically significant alterations of any lymphocyte subpopulations were observed for ER stimulated spleen cell cultures. In PBMC cultures (Additional file 9B), CD4+ cells comprised the largest population identified at approximately 20% followed by TCRγ/δ−CD8αβ+ cells at approximately 10% and TCRγ/δ+CD8− cells at approximately 2% while the remaining three populations were markedly smaller at less than 0.5% each. In general, for PBMC cultured in medium without ER antigen the proportions of all identified lymphocyte subpopulations were lower in cultures from L10L chickens compared to those from L10H chickens and some of these differences were statistically significant. Moreover, in PBMC cultures from both infected and uninfected L10H and L10L chickens stimulated with ER antigen the proportions of all identified lymphocyte subpopulations tended to be increased compared to those in corresponding medium cultures and some of these differences were statistically significant.

**Figure 5:**
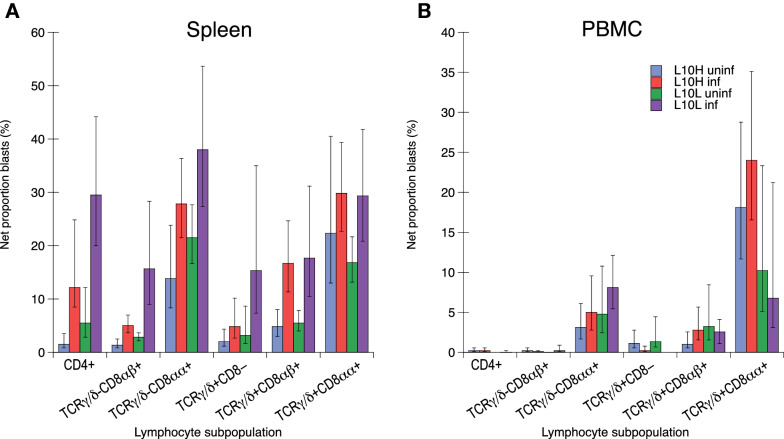
**Net proportions of blast transformed cells in different lymphocyte subpopulations upon in vitro ER antigen stimulation for 72 h.** Results for cultures of leukocytes obtained from uninfected L10H (uninf; blue bars) or L10L (uninf; green bars) chickens or experimentally ER infected L10H (inf; red bars) or L10L (inf; purple bars) chickens. Chickens were infected on day 0 and spleens (**A**) and PBMC (**B**) were collected on day 18 after infection. Values are group means ± 95% CI (9 ≥ n ≤ 12) where non-overlapping CI indicate statistically significant differences. Note that CI for net proportions are asymmetrical, for details see Materials and methods. Lymphocyte subpopulations were identified using monoclonal antibody panel 3 (Table 2) and the gating strategy is described in Additional file 3.

The proportion of spontaneously blast-transformed cells after 72 h of culture in growth medium alone varied between spleen cells and PBMC as well as the different lymphocyte subpopulations studied (Additional files 9C and D). Overall, the highest proportions of blast-transformed cells were observed in spleen cell cultures where TCRγ/δ−CD8αα+ and TCRγ/δ+CD8αα+ cells showed the highest spontaneous blast transformation and TCRγ/δ−CD8αβ+ the lowest. In PBMC cultures, TCRγ/δ−CD8αα+ cells also showed the highest spontaneous blast transformation while CD4+ and TCRγ/δ−CD8αβ+ cells showed the lowest. For both spleen cells and PBMC, cultures from L10L chickens tended to have higher proportions of spontaneous blast transformed cells in most identified lymphocyte subpopulations compared to cultures from L10H chickens. Moreover, for all lymphocyte subpopulations a large variation in spontaneous blast transformation was observed between individuals. Therefore, blast transformation in ER antigen stimulated cultures was analysed as net proportions of blast transformed cells (i.e. % blast transformed cells in ER stimulated cultures minus % blast transformed cells in medium cultures) within each chicken for each lymphocyte subpopulation to reduce the variation influenced by individual (Figure 5). Results showed that in spleen cell cultures (Figure 5A), blast transformation of CD4+ and TCRγ/δ−CD8αβ+ cells induced upon ER antigen stimulation was statistically significantly higher in cultures from ER infected chickens from both L10 subgroups compared to that in cultures from corresponding uninfected chickens. The net proportions of ER induced blast transformation were higher for CD4+ cells compared to TCRγ/δ−CD8αβ+ cells and for both these cell populations proliferative responses were higher in cultures from L10L chickens compared to those from L10H chickens. Moreover, ER antigen stimulation also induced statistically significant higher blast transformation of TCRγ/δ+CD8αβ+ cells in cultures from ER infected chickens from both L10 subgroups compared to that in cultures from corresponding uninfected chickens. For this cell population the ER induced proliferative responses were similar in cultures from L10L and L10H chickens. For TCRγ/δ−CD8αα+, TCRγ/δ+CD8− cells and TCRγ/δ+CD8αα+ spleen cells ER antigen stimulation induced blast transformation in cultures from both uninfected and ER infected chickens without significant differences between uninfected and infected chickens or between L10H and L10L chickens. In PBMC cultures (Figure 5B), very low or no blast transformation of CD4+ and TCRγ/δ−CD8αβ+ cells was induced by ER antigen stimulation. In these cultures, the most prominent proliferative responses to ER antigen stimulation were observed for TCRγ/δ−CD8αα+ and TCRγ/δ+CD8αα+ cells, and lower responses were also observed for TCRγ/δ+CD8− and TCRγ/δ+CD8αβ+ cells. None of these responses were significantly different in cultures from uninfected or infected chickens or from L10H or L10L chickens, respectively.

Thus, ER antigen specific in vitro activation of CD4+, TCRγ/δ−CD8αβ+ and TCRγ/δ+CD8αβ+ T-cell subsets was observed for spleen cells collected 18 days after ER infection of chickens. In addition, ER antigen stimulation also elicited unspecific (i.e. in cells from both uninfected and infected chickens) proliferative responses particularly in spleen and PBMC TCRγ/δ−CD8αα+ and TCRγ/δ+CD8αα+ in both L10 subgroups.

### Systemic ER-specific IgM and IgY responses

**Figure 6:**
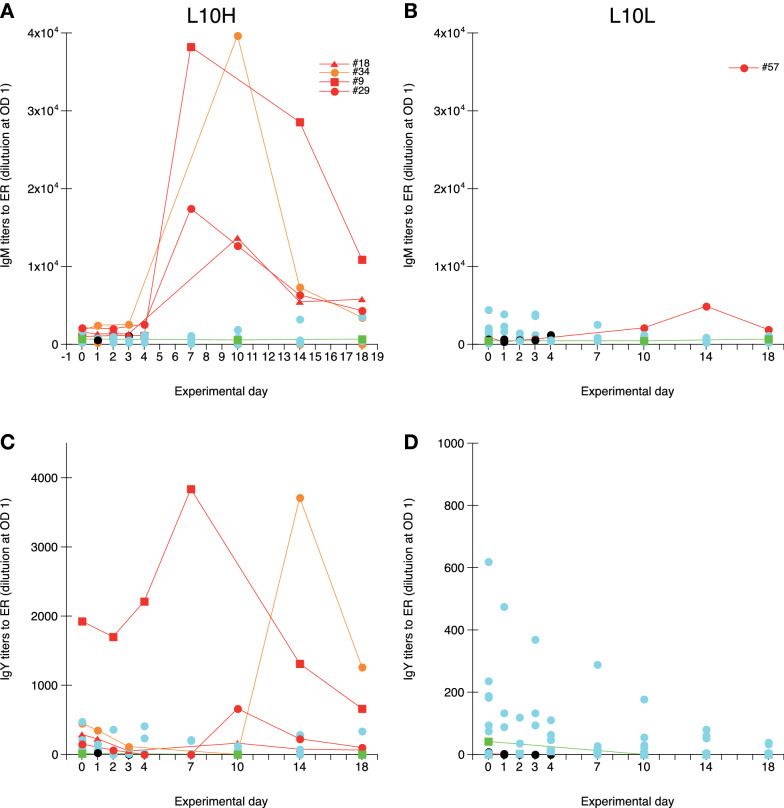
**IgM (A, B) and IgY (C, D) titers to ER in serum.** Results from L10H (**A**, **C**) and L10L (**B**, **D**) chickens at the indicated days after ER infection on day 0. Results are individual values for infected chickens and geometric mean values ± 95% CI for uninfected chickens (green squares). Black circles: chickens that eventually died, red circles: chickens that showed clear clinical signs of disease at one or more occasions, orange circles: chickens positive for ER in blood at one or more occasions without clear clinical signs of disease, light blue circles: infected chickens without clinical signs of disease or bacteraemia.

Quantification of IgM and IgY titers to ER was performed on all serum samples collected from the ER infected chickens and on serum samples collected on days 0, 10 and 14 from chickens in the uninfected control groups (Figure 6). For IgM titers to ER, clear transient increases in titers, with peak titers on day 7 or 10, were observed for all three surviving L10H chickens that showed clinical signs at one or more occasions during the experiment and one of the bacteraemic L10H chickens without clear clinical signs (Figure 6A). For the one surviving L10L chicken with bacteraemia a trend to increased ER IgM titers was indicated on day 14 (Figure 6B), although this increase was very low in comparison to the responses observed for L10H chickens. None of the other ER infected L10H or L10L chickens showed any increases in IgM levels to ER. For IgY titers to ER, high transient increases in titers, with peak titers on day 7 or 14, were observed for the two L10H chickens with the highest IgM responses to ER (Figure 6C). One of these chickens also had the highest putatively maternally derived pre-infection IgY titers to ER on day 0. One of the L10H chickens with a clear but lower IgM response to ER also showed a trend to increased IgY titers to ER with peak titers on day 10, while the remaining L10H chicken positive for IgM to ER did not show any increases in IgY to ER. None of the remaining ER infected L10H chickens or any of the ER infected L10L chickens (Figure 6D) showed any signs of IgY responses to ER.

Hence, some ER infected L10H chickens with clinical signs and/or bacteraemia showed clear IgM responses to ER and some of these L10H chickens also showed transient IgY responses to ER.

### Determination of MHC genotype

The MHC genotype for each chicken included in the experiment was determined by LEI0258 genotyping. The results (Table 1) showed that all L10L chickens and 17 of the L10H chickens (10 infected and 7 uninfected) were homozygous 193 bp (BW3-like) haplotype and the remaining 11 L10H chickens were heterozygous 193 bp/448 bp haplotype (4 infected and 7 uninfected). Two of the infected homozygous 193 bp and 5 of the infected 193 bp/448 bp heterozygous L10H chickens showed clinical signs and/or bacteraemia, i.e. 50% of both MHC genotypes (Additional file 4).

## Discussion

The present study was undertaken to gain more insights into chicken immune responses to ER infection. The most striking finding was the identification of ER specific T-cell activation upon in vitro re-stimulation of spleen cells. To our knowledge, this is the first report on ER specific T-cell activation in the chicken. In other species, ER specific proliferation of lymphocytes induced upon in vitro antigen re-stimulation has been observed in two studies using porcine cells [46, 47]. In these studies, proliferation of PBMC was reported 2 to 10 weeks after vaccination with a live ER vaccine [47], or 2 weeks after vaccination with an inactivated ER bacterin preparation and 8 days after intradermal ER infection of pigs [46]. In the former study, in vitro proliferative responses observed after vaccination were associated with better protection after challenge infection compared to other tested vaccine preparations [46]. In the present study, we only observed ER-specific T-cell proliferation in cultures of spleen cells and not in PBMC. In addition to these results from day 18 after infection we also performed a pilot ER antigen stimulation of spleen cells and PBMC from the four L10H chickens, 2 infected and 2 uninfected, sacrificed on day 7 after infection (data not shown) where no antigen specific T-cell proliferation was observed. At day 18 after infection, we observed proliferative responses of CD4+ spleen T-cells. The CD4+ cells in this dataset contain both CD4+CD8− cells that presumably comprise the chicken “classical” T-helper cells as well as CD4+CD8α+ cells [48, 49]. However, the inbred L10H and L10L chickens have very few CD4+CD8αα+ cells among PBMC and spleen cells [50]. It was therefore not meaningful to analyse for these cells separately and hence the CD4+ cells in the present material may comprise T-helper cells with potentially slightly different functions e.g. as putative effector/memory cells. Nonetheless, upon in vitro antigen re-stimulation it is expected to find T-helper cells among the responding cells and without further identification of features such as cytokine production profiles it is not possible to define the type of Th-response, e.g. as a Th1- or Th2-type response. However, the present ER antigen stimulation also induced recall responses of TCRγ/δ−CD8αβ+ cells, i.e., CTL, that constitute a key cell in Th1-type responses. Hence, this observation suggests that a Th1-type T-cell response was induced in the chickens by the ER infection. Similarly, a study of in vitro ER antigen induced cytokine mRNA responses in PBMC from Atlantic bottlenose dolphins (*Tursiops truncatus*) showed that antigen specific interferon (IFN)-γ expression was induced after vaccination with an ER sub-unit vaccine for pigs and/or natural ER infection [51]. In a host, ER bacteria are often located intracellularly [16, 33, 52–54] and it has for instance been shown that ER may survive and replicate in murine macrophages [16, 53]. It is therefore likely that a Th1-type response specialised for intracellular pathogens and comprising e.g., IFN-γ mediated activation of macrophages is effective to mediate protective immunity against ER infection. In analogy, the clearance of the intra-macrophage bacterial pathogen *Salmonella* by the chicken immune system is considered dependent on Th1-type responses and IFN-γ production [15].

Interestingly, in addition to the “classical” TCRα/β expressing T-cells we also found evidence for ER antigen specific activation of TCRγ/δ+CD8αβ+ T-cells in the spleen cell cultures. The TCRγ/δ expressing T-cells constitute a substantial population of both PBMC and spleen cells in the chicken but despite their relative abundance many aspects of their role in the chicken immune system are still unknown. Nonetheless, it has been shown they may be recruited and/or activated upon infection of chickens with e.g. *Salmonella* [55–57], Marek’s disease virus [58] and the protozoan parasite *Eimeria maxima* [59], that they have cytolytic capacity [60] and that they can be IFN-γ producers during early infection responses [56, 58]. To date, we are not aware of any clear evidence of antigen specific TCRγ/δ+ T-cell memory responses in the chicken and the TCRγ/δ+CD8αβ+ cell responses observed herein may also be due to bystander activation through the TCRα/β+ T-cell responses in these cultures. However, in mammals antigen specific TCRγ/δ expressing T-cells have been identified for instance in bacterial infections in humans, non-human primates, mice and cattle [61, 62].

In addition to ER antigen specific responses, we also observed unspecific, i.e., both in uninfected and infected chickens, blast transformation induced by ER stimulation both in spleen cell and PBMC cultures particularly in TCRγ/δ+CD8αα+ and TCRγ/δ−CD8αα+ cells. The TCRγ/δ+CD8αα+ T-cell subpopulation has been identified as a prominent innate responding cell during early *S. enterica* infection e.g. by expressing IFN-γ [56], and among chicken TCRγ/δ+ T-cell subpopulations they uniquely express Toll-like receptor 4 mRNA [63]. Moreover, upon stimulation of chicken whole blood cultures with live *S. enterica* increased CD25 expression was observed for TCRγ/δ expressing T-cells with the highest CD25 expression levels on the TCRγ/δ+CD8αα+ subpopulation [57]. Hence, the ER induced blast transformation observed in the present study may reflect that TCRγ/δ expressing T-cells have a role also in the early recognition of ER infection in the chicken. In addition, the TCRγ/δ−CD8αα+ cells that were also activated upon ER stimulation may comprise several cell types including innate cells such as NK-cells [64] and the unspecific blast transformation observed herein may thus indicate that other innate lymphoid cells are similarly involved in early responses to ER infection.

The ER specific blast transformation responses of CD4+ and TCRγ/δ−CD8αβ+ cells were of statistically significantly higher magnitude in cells from the L10L chickens compared to those from the L10H chickens. In comparison, an inverse relationship between serum MBL levels and specific antibody titers has been reported in chickens [22, 65] and in mice [66, 67]. It was then hypothesised that this might be due to higher MBL levels leading to more rapid clearance of the pathogen/antigen early after infection or vaccination and therefore less antigen available for initiation of specific antibody responses. Higher antigen specific antibody responses have also been observed for L10L chickens compared to L10H chickens in some experiments [32] but not consistently [23, 24, 68]. The argument on antigen availability could likewise be put forward to explain the present T-cell results. However, during the selection process to achieve the current differences in serum MBL levels the chicken lines may also unintentionally have acquired genetic differences in other traits including those for immune functions. We have for instance found that mRNA expression of collectin genes found located in close proximity to the MBL gene at chicken chromosome 6, namely lung lectin (cLL) and surfactant protein A (SPA), in the upper respiratory tract was higher in 3 week old L10H chickens compared to age matched L10L chickens (unpublished observation). Moreover, in a study of spleen transcriptomes during IBV infection of L10 chickens significant differences in gene expression involved in the activation of lymphocytes between L10H and L10L chickens were observed both in uninfected chickens and after IBV infection, e.g. enrichment of Gene Ontology terms “Lymphocyte activation involved in immune response” in uninfected chickens and “Alpha-beta T cell activation” in IBV infected chickens [69]. In the present study we in fact noticed a general trend of higher spontaneous blast transformation for L10L cells of most of the lymphocyte subpopulations studied, which might reflect a higher activation potential of L10L lymphoid cells compared to that of L10H cells. Interestingly though, such a trend was not clearly obvious for the ER induced responses of all studied lymphoid subpopulations. Moreover, PBMC isolated from L10H chickens showed higher responses upon in vitro stimulation with polyclonal mitogen Con A compared to PBMC from L10L chickens [70]. Hence, further work is needed to fully characterise the genetic influence on immune responses in these chickens.

The primary intention for this experiment was to study the role of MBL in the initial innate immune responses against ER infection using the two L10 chicken sublines. Unfortunately, the clinical outcome of this experimental ER infection was very varied and did not allow for any clear conclusions to be drawn on the influence of MBL levels on the outcome and progression of ER infection. The current results with low numbers of inoculated chickens displaying bacteraemia are similar to what we have observed in previous studies when using ER infection doses of the current strain between 0.5 × 10^5^ and 1.6 × 10^8^ cfu ER/chicken [33]. We aimed at infecting the current chickens with approximately 10^10^ cfu ER/chicken since this dose previously gave 100% of chickens positive for ER in blood samples collected on day 3 after infection [33]. Regrettably, this was not achieved despite identical bacterial culture procedures, and it seems likely that the inadvertently low infection dose of 0.75 × 10^8^ ER/chicken in the present infection was the reason for the low recovery rate of ER in blood samples. Nonetheless, in the present experiment individual chickens with high levels of bacteraemia and severe clinical signs as well as individuals displaying clinical signs and bacteraemia over a longer period, up to days 7 and 10 after infection, respectively, were observed. This was unexpected and differed from our previous experience with this ER strain [33]. Among the few other experimental ER infections of chickens described in the literature [1, 11, 12, 71, 72] high infection doses, 10^9^–10^10^ cfu ER, have been used with varying outcome, from 100% mortality to no clinical signs, and varying recovery of bacteria even when identical ER strains have been used. Thus, other factors than ER strain and the infection dose such as host traits including genetic background and management likely affect both the level of bacteraemia and nature of clinical signs in ER infected chickens.

Unsurprisingly considering the varied clinical outcome of the current ER infection, immune responses observed also showed a large variation between individuals. Prompt blood heterophil, monocyte and MBL responses were observed in both groups of ER infected chickens in analogy with what we have previously observed upon ER infection [33] and these responses were more pronounced for chickens with bacteraemia and/or clinical signs. Chickens with prolonged bacteraemia and clinical signs were only observed in the L10H group and hence it seemed that high serum MBL levels did not categorically lead to a more rapid clearance of ER from the circulation but it should be stressed that there were too few observations in the current experiment to draw any firm conclusions.

Interestingly, the most consistent sign of early immune activation observed in all ER infected chickens in the current study was the increased expression of mannose receptor MRC1L-B and concurrent decreased expression of MHCII on circulating monocytes. Increased MRC1L-B expression was likewise observed on monocytes day 1 after ER infection of chickens in our previous study [33]. Up regulation of MRC1L-B expression has also been observed on chicken mononuclear phagocytes upon in vitro stimulation with innate defence peptides [73]. Moreover, a transient decrease of MHCII expression on peripheral blood monocytes and concurrent increased phagocytic activity of monocytes 4 days after infection of chickens with the gram-negative bacterium *Avibacterium paragalliarum* has been reported [74]. In a study of chicken splenic macrophages two distinct populations MRC1L-B^high^MCHII^low^ and MRC1LB^low^MHCII^high^, respectively, were identified [75] and it was shown that the MRC1L-B^high^MCHII^low^ population increased upon intra-peritoneal LPS challenge. Furthermore, the MRC1LB^high^MCHII^low^ population showed higher phagocytic capacity, higher migratory capacity, lower antigen presenting properties and lower expression of pro-inflammatory cytokines interleukin (IL)-1β, IL-6 and IL-12 compared to the MRC1LB^low^MHCII^high^ population. Thus, in this context our present and previous results suggests that ER infection quickly induces either alteration of cell surface expression on monocytes or re-distribution of monocyte populations in the circulation that might favour a rapid phagocytic clearance of the infection.

Unlike in our previous ER infections of chickens, no or very inconsistent and brief specific antibody responses were observed in the present study. We have no obvious explanation for this since in previous ER infection experiments chickens have shown clear production of IgY specific to ER with a trend that chickens infected with lower infection doses produced higher antibody levels [41]. The low antibody responses observed in the present study could be due to genetic differences between L10 chickens and the conventional layer hybrids previously used. Genetic differences in antibody production for instance elicited by vaccination have been described for chickens [76, 77]. However, both L10H and L10L chickens have readily responded with antigen specific IgY production upon IBV vaccination and/or infection [23, 24, 32, 68].

In conclusion, results from the present study showed induction of ER-specific T-cell responses among CD4+, CTL and TCRγ/δ+CD8αβ+ cells. This indicates that a Th1-type response was initiated by the infection, which may be important to clear intracellular ER bacteria. In addition, findings on in vitro innate ER activation of TCRγ/δ+CD8αα+ cells and rapid changes in circulating monocytes to MRC1L-B^high^MCHII^low^ expressing cells upon ER infection indicate that early innate recognition of ER may be directed to phagocytic activation prior to Th1-induction.

## Supplementary Information

**Additional file 1. Gating strategy for flow cytometry using panel 1.** Identification of counting beads, heterophils, monocytes, thrombocytes, and B-cells and MHCII expression on B-cells and monocytes through singlet gating, FSC/SSC characteristics and using CD45-PE/Cy7, CD41/61-Fitc, KUL01-RPE (MRC1L-B), Bu-1-APC/Cy7 and MHCII-PerCp/Cy5.5. From the gate R1 of very high SSC events in initial dot-plot in (A) counting beads were identified as high fluorescent in (B). From all events in (A) gating through FSC-H vs. FSC-A was performed in (C) to identify singlets. From this gate high CD45 expressing events (leukocytes) and low SCC-A and medium to high CD45 expressing events (potential thrombocytes) were gated in (D). From the CD45 gate events were defined according to CD41/61 expression in (E) and high CD41/61 events were defined according to FSC and SSC characteristics as thrombocytes in (F). Low CD41/61 expressing events in (E) were defined according to MRC1L-B and MHCII expression in (G) and MRC1L-B+MHCII+ events were defined according to FSC and SSC characteristics as monocytes in (F). Low MRC1L-B events in (G) were defined according to Bu-1 and MHCII expression in (H) where Bu-1+MHCII+ events were defined as B-cells. Low MRC1L-B events in (G) were defined according to FSC and SSC characteristics in (I) where high SSC events were defined as heterophils. A representative blood sample from an uninfected chicken L10H chicken on day 10 is shown. The antibody panel is described in Table 2.

**Additional file 2. Gating strategy for flow cytometry using panel 2.** Identification of counting beads, TCRγ/δ+, CD4+, CD4−CD8αβ+ (CTL) and CD4−CD8αα+ cells and CD25 expression on these through singlet gating, FSC/SSC characteristics and using TCRγ/δ−PerCp/Cy5.5, CD4−Fitc, CD8α−Cy5, CD8β−RPE, and CD25−APC/Cy7. From the gate R1 of very high SSC events in initial dot-plot in (A) counting beads were identified as high fluorescent in (B). From all events gating through FSC-H vs. FSC-A was performed in (C) to identify singlets. Singlets were defined as “lymphocytes” according to FSC and SSC characteristics in (A). “Lymphocytes” were defined according to TCRγ/δ expression as TCRγ/δ+ in (D) and CD25 expression on TCRγ/δ+ was defined in (E). Non-TCRγ/δ+ events in (D) were defined according to CD4 and CD8α expression in (F) and CD4+ cells were identified (this gate also comprises CD4+CD8α+ cells in some individuals, like the one shown here, as mentioned in the Discussion). CD25 expression on CD4+ cells was defined in (G). CD4−CD8α+ cells identified in (F) were defined according to CD8β expression in (H) and CD4−CD8αβ+ (CTL) and CD4−CD8αα+ cells were identified. CD25 expression on CD4−CD8αβ+ (CTL) and CD4−CD8αα+ cells was defined in (I). A representative blood sample from an uninfected L10H chicken on day 10 is shown. The antibody panel is described in Table 2.

**Additional file 3. Gating strategy for flow cytometry using panel 3.** Identification of CD4+, TCRα/β+CD8αβ+ (CTL), TCRγ/δ−CD8αα+, TCRγ/δ+CD8αβ+, TCRγ/δ+CD8αα+TCRγ/δ+CD8− cells and blast transformation, respectively, of these cells through singlet gating, FSC/SSC characteristics and using CD4-PACBLU, TCRγ/δ-Fitc, CD8β-RPE, and CD8α-Cy5. From the gate excluding debris in initial dot-plot in (A) wide gating through FSC-H vs. FSC-A was performed in (B) to include potential blast and exclude large aggregates. From this gate live cells were gated through exclusion of events stained with Aqua dead stain in (C). From the live gate CD4+ events were gated in (D). From the live gate events were defined according to TCRγ/δ expression in (E). TCRγ/δ+ events defined in (E) were further defined into TCRγ/δ+CD8αβ+, TCRγ/δ+CD8αα+TCRγ/δ+CD8− in (F). TCRγ/δ− events defined in (E) were further defined as TCRγ/δ−CD8αβ+ (CTL) and TCRγ/δ−CD8αα+ in (G) (the TCRγ/δ−CD8− gate was not used in the analysis). All defined cell populations were examined for blast transformation (FSC and SSC high) as exemplified for CD4+ cells in (H). A representative spleen cell sample cultured in growth medium without additives from an uninfected chicken on day 18 is shown. The antibody panel is described in Table 2.

**Additional file 4. Clinical signs, ER re-isolation and** ***post-mortem*** **findings of chickens positive for ER in blood.**

**Additional file 5. Body weights (A, B) and daily weight gains (C, D).** Results for L10H chickens (A, C) and L10L (B, D) chickens at the indicated experimental days. Values are group means ± 95% CI for chickens infected with ER on day 0 (dark blue symbols) and for uninfected chickens (green symbols), where non-overlapping CI indicate statistically significant differences, and for individual chickens with clinical signs of disease at one or more occasions (red symbols) and chickens positive for ER in blood at one or more occasions without clinical signs of disease (orange symbols).

**Additional file 6. Numbers of thrombocytes in blood.** Results from (A) L10H and (B) L10L chickens at the indicated days after ER infection on day 0. Results are mean values ± 95% CI for infected chickens (dark blue circles) and uninfected chickens (green squares), where non-overlapping CI indicate statistically significant differences, and individual values for infected chickens. Black circles: chickens that eventually died, red circles: chickens that showed clear clinical signs of disease at one or more occasions, orange circles: chickens positive for ER in blood at one or more occasions without clear clinical signs of disease, light blue circles: infected chickens without clinical signs of disease or bacteraemia.

**Additional file 7. Numbers of different lymphocyte subpopulations.** B-cells (A, B); TCRγ/δ+ cells (C, D); CD4+CD8− cells (E, F); CD4−CD8αβ+ cells (G, H) and CD4−CD8αα+ cells (I, J), in blood from L10H (A, C, E, G, I and K) and L10L (B, D, F, H, J and L) chickens at the indicated days after ER infection on day 0. Results are mean values ± 95% CI for infected chickens (dark blue circles) and uninfected chickens (green squares), where non-overlapping CI indicate statistically significant differences, and individual values for infected chickens. Black circles: chickens that eventually died, red circles: chickens that showed clear clinical signs of disease at one or more occasions, orange circles: chickens positive for ER in blood at one or more occasions without clear clinical signs of disease, light blue circles: infected chickens without clinical signs of disease or bacteraemia. Monoclonal antibody panels for immunolabelling are described in Table 2 and gating strategies in Additional files 1 and 2.

**Additional file 8. Proportions of CD25 expressing cells of different lymphocyte subpopulations.** CD4+CD8− cells (A, B); CD4−CD8αβ+ cells (C, D); and TCRγ/δ+ cells (E, F), in blood from L10H (A, C, and E) and L10L (B, D and F) chickens at the indicated days after ER infection on day 0. Results are mean values ± 95% CI for infected chickens (dark blue circles) and uninfected chickens (green squares), where non-overlapping CI indicate statistically significant differences, and individual values for infected chickens. Black circles: chickens that eventually died, red circles: chickens that showed clear clinical signs of disease at one or more occasions, orange circles: chickens positive for ER in blood at one or more occasions without clear clinical signs of disease, light blue circles: infected chickens without clinical signs of disease or bacteraemia. Monoclonal antibody panels for immunolabelling are described in Table 2 and gating strategies in Additional file 2.

**Additional file 9. Proportions of the indicated lymphocyte subpopulations.** Results are proportions out of live cells in spleen cells (A) or PBMC (B) from uninfected (uninf; blue bars) or ER infected (inf; red bars) L10H chickens or uninfected (uninf; green bars) or ER infected (inf; purple bars) chickens, respectively, cultured for 72 h in growth medium alone (medium; dark coloured bars) or growth medium supplemented with ER antigen (ER; light coloured bars). Spleens and blood samples were collected on day 18 after ER infection of chickens and values are group means ± 95% CI (9 ≥ n ≤ 12), where non-overlapping CI indicate statistically significant differences. Proportions of spontaneously blast transformed cells in cultures of spleen cells (C) or PBMC (D) collected on day 18 after ER infection out of the indicated lymphocyte subpopulations after 72 h of culture in growth medium alone. Individual values of all L10H (H) or L10L (L) chickens included are shown as open circles and mean values for the indicated lymphocyte subpopulations are represented by a line. Lymphocyte subpopulations were identified using monoclonal antibody panel 3 (Table 2) and the gating strategy is described in Additional file 3.

## Data Availability

All results from the study are included in this published article and its Additional files and raw data are available from the corresponding author on reasonable request.
