# Supplementary figures and images for: Erysipelothrix rhusiopathiae-specific T-cell responses after experimental infection of chickens selectively bred for high and low serum levels of mannose-binding lectin
Source: Vet Res. 2022 Dec 12;53:105. doi: 10.1186/s13567-022-01126-w (PMC9743643; doi:10.1186/s13567-022-01126-w)

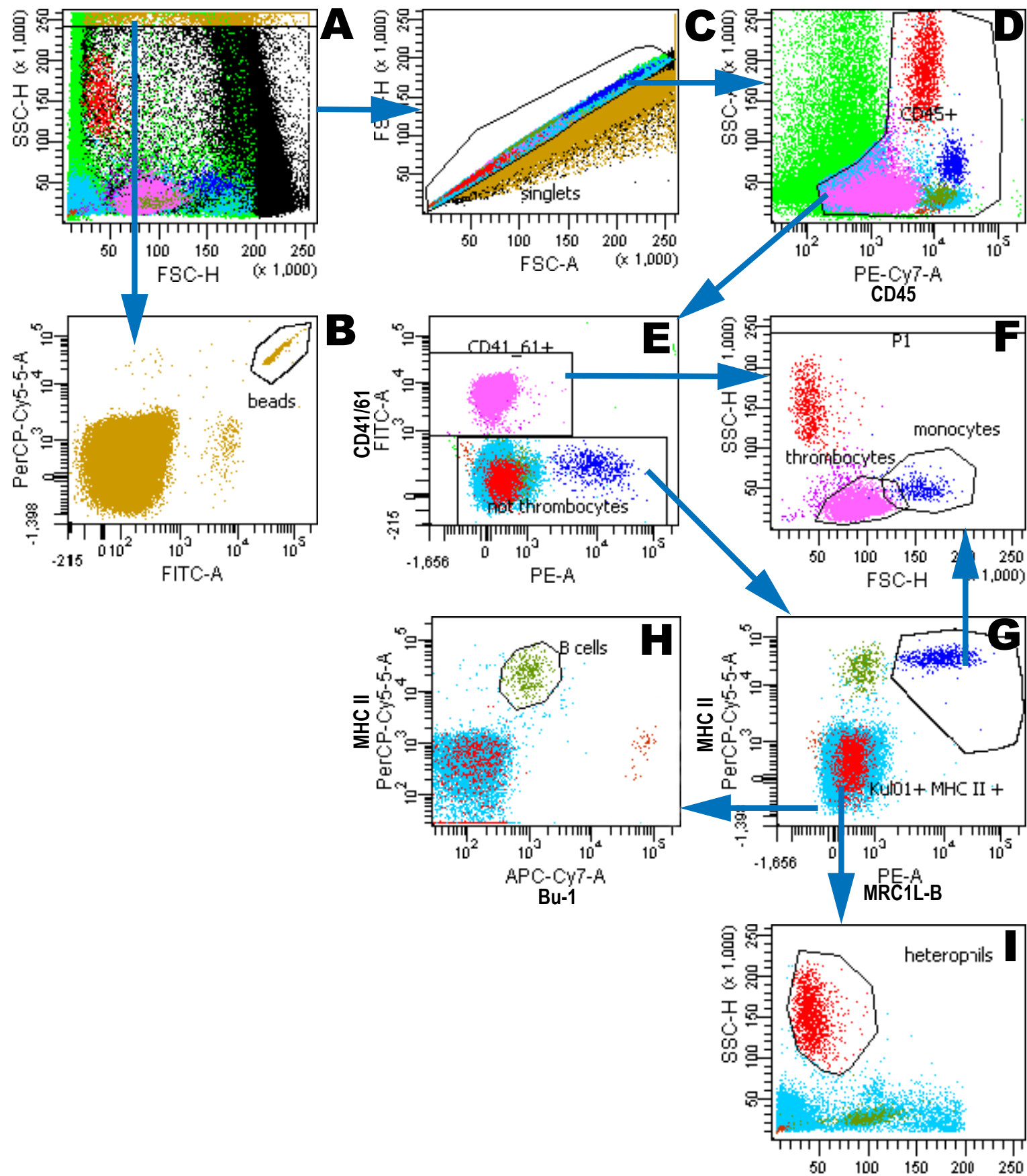

Supplement: Supplementary file 1 — Additional file 1. Gating strategy for flow cytometry using panel 1. Identification of counting beads, heterophils, monocytes, thrombocytes, and B-cells and MHCII expression on B-cells and monocytes through singlet gating, FSC/SSC characteristics and using CD45-PE/Cy7, CD41/61-Fitc, KUL01-RPE (MRC1L-B), Bu-1-APC/Cy7 and MHCII-PerCp/Cy5.5. From the gate R1 of very high SSC events in initial dot-plot in (A) counting beads were identified as high fluorescent in (B). From all events in (A) gating through FSC-H vs. FSC-A was performed in (C) to identify singlets. From this gate high CD45 expressing events (leukocytes) and low SCC-A and medium to high CD45 expressing events (potential thrombocytes) were gated in (D). From the CD45 gate events were defined according to CD41/61 expression in (E) and high CD41/61 events were defined according to FSC and SSC characteristics as thrombocytes in (F). Low CD41/61 expressing events in (E) were defined according to MRC1L-B and MHCII expression in (G) and MRC1L-B+MHCII+ events were defined according to FSC and SSC characteristics as monocytes in (F). Low MRC1L-B events in (G) were defined according to Bu-1 and MHCII expression in (H) where Bu-1+MHCII+ events were defined as B-cells. Low MRC1L-B events in (G) were defined according to FSC and SSC characteristics in (I) where high SSC events were defined as heterophils. A representative blood sample from an uninfected chicken L10H chicken on day 10 is shown. The antibody panel is described in Table 2. [file 13567_2022_1126_MOESM1_ESM.pdf]

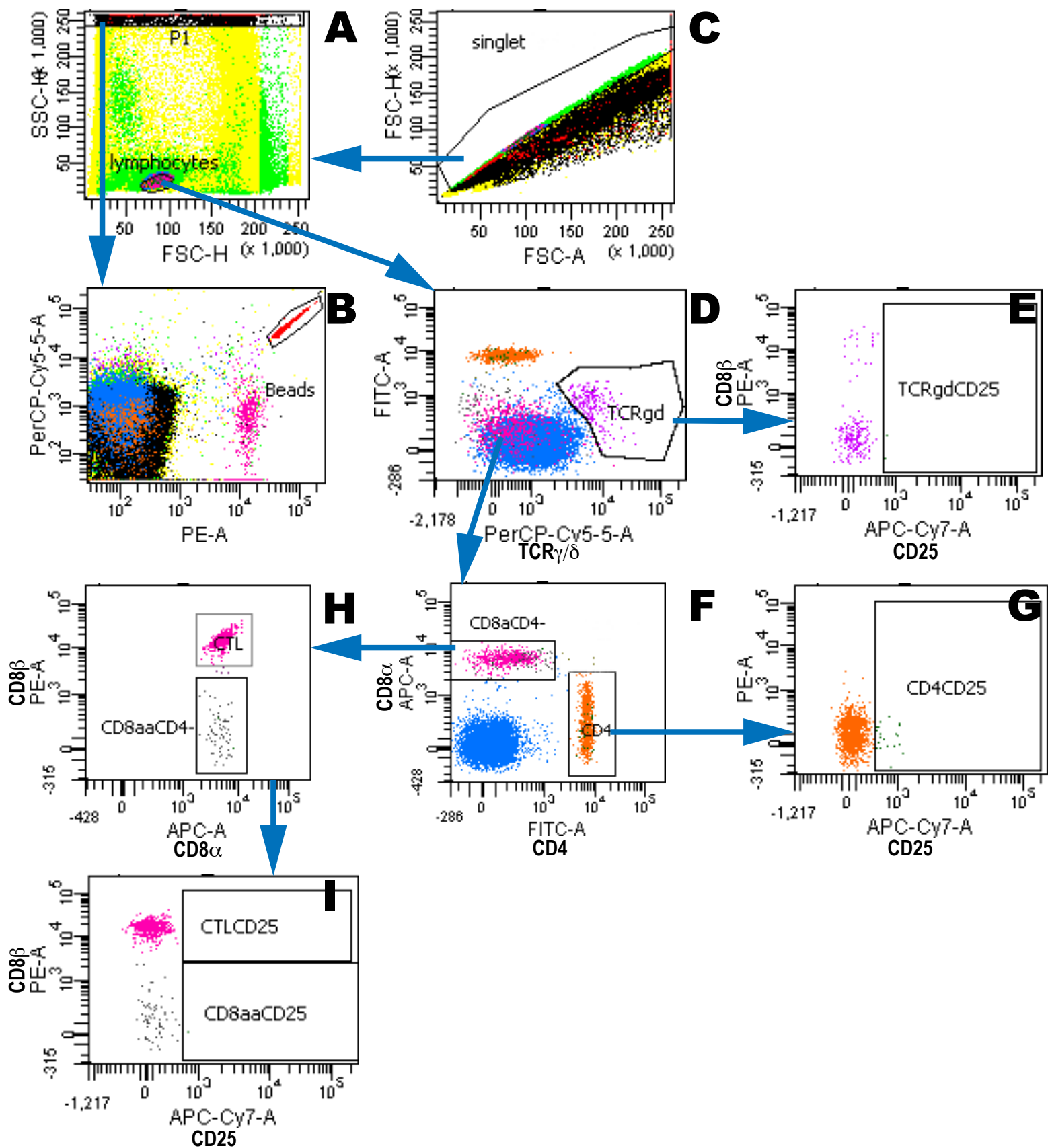

Supplement: Supplementary file 2 — Additional file 2. Gating strategy for flow cytometry using panel 2. Identification of counting beads, TCRγ/δ+, CD4+, CD4−CD8αβ+ (CTL) and CD4−CD8αα+ cells and CD25 expression on these through singlet gating, FSC/SSC characteristics and using TCRγ/δ−PerCp/Cy5.5, CD4−Fitc, CD8α−Cy5, CD8β−RPE, and CD25−APC/Cy7. From the gate R1 of very high SSC events in initial dot-plot in (A) counting beads were identified as high fluorescent in (B). From all events gating through FSC-H vs. FSC-A was performed in (C) to identify singlets. Singlets were defined as “lymphocytes” according to FSC and SSC characteristics in (A). “Lymphocytes” were defined according to TCRγ/δ expression as TCRγ/δ+ in (D) and CD25 expression on TCRγ/δ+ was defined in (E). Non-TCRγ/δ+ events in (D) were defined according to CD4 and CD8α expression in (F) and CD4+ cells were identified (this gate also comprises CD4+CD8α+ cells in some individuals, like the one shown here, as mentioned in the Discussion). CD25 expression on CD4+ cells was defined in (G). CD4−CD8α+ cells identified in (F) were defined according to CD8β expression in (H) and CD4−CD8αβ+ (CTL) and CD4−CD8αα+ cells were identified. CD25 expression on CD4−CD8αβ+ (CTL) and CD4−CD8αα+ cells was defined in (I). A representative blood sample from an uninfected L10H chicken on day 10 is shown. The antibody panel is described in Table 2. [file 13567_2022_1126_MOESM2_ESM.pdf]

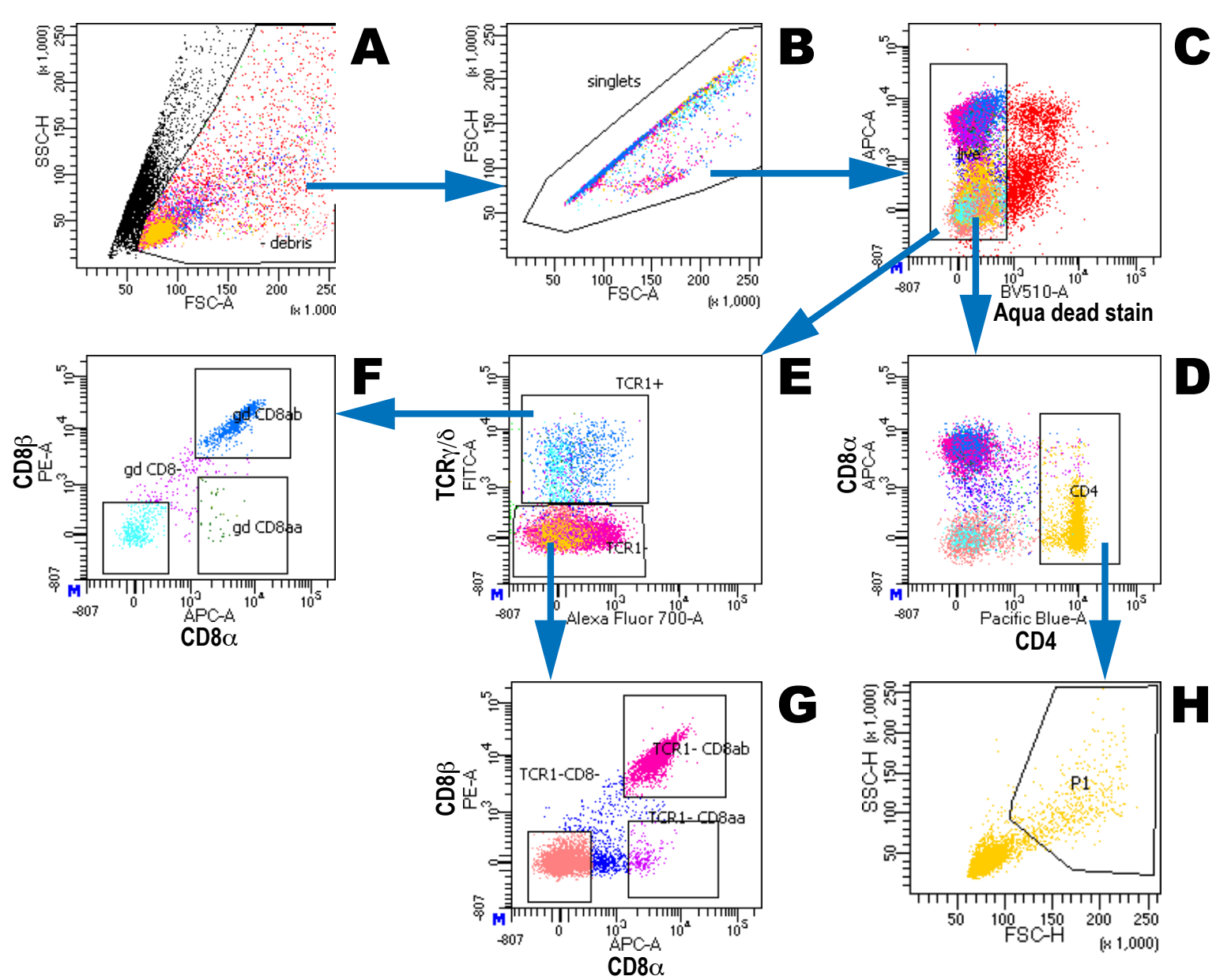

Supplement: Supplementary file 3 — Additional file 3. Gating strategy for flow cytometry using panel 3. Identification of CD4+, TCRα/β+CD8αβ+ (CTL), TCRγ/δ−CD8αα+, TCRγ/δ+CD8αβ+, TCRγ/δ+CD8αα+TCRγ/δ+CD8− cells and blast transformation, respectively, of these cells through singlet gating, FSC/SSC characteristics and using CD4-PACBLU, TCRγ/δ-Fitc, CD8β-RPE, and CD8α-Cy5. From the gate excluding debris in initial dot-plot in (A) wide gating through FSC-H vs. FSC-A was performed in (B) to include potential blast and exclude large aggregates. From this gate live cells were gated through exclusion of events stained with Aqua dead stain in (C). From the live gate CD4+ events were gated in (D). From the live gate events were defined according to TCRγ/δ expression in (E). TCRγ/δ+ events defined in (E) were further defined into TCRγ/δ+CD8αβ+, TCRγ/δ+CD8αα+TCRγ/δ+CD8− in (F). TCRγ/δ− events defined in (E) were further defined as TCRγ/δ−CD8αβ+ (CTL) and TCRγ/δ−CD8αα+ in (G) (the TCRγ/δ−CD8− gate was not used in the analysis). All defined cell populations were examined for blast transformation (FSC and SSC high) as exemplified for CD4+ cells in (H). A representative spleen cell sample cultured in growth medium without additives from an uninfected chicken on day 18 is shown. The antibody panel is described in Table 2. [file 13567_2022_1126_MOESM3_ESM.pdf]

**L10H****A**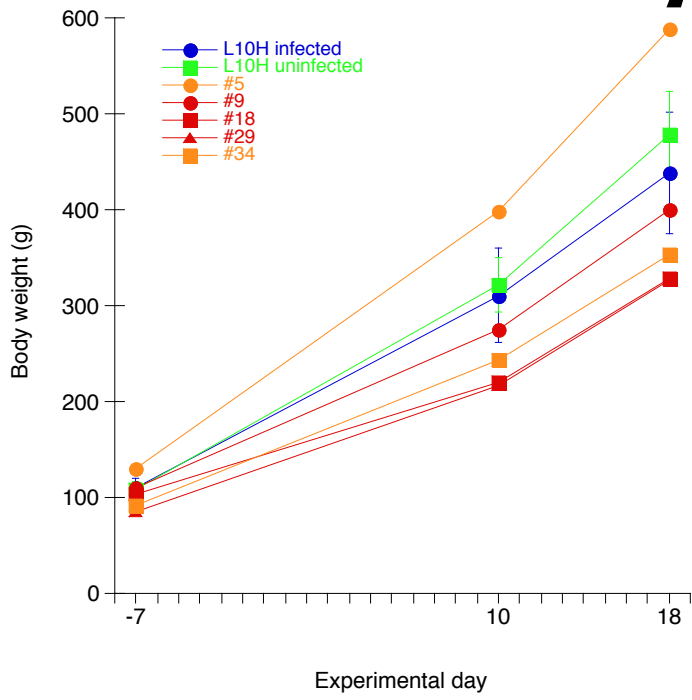**L10L****B**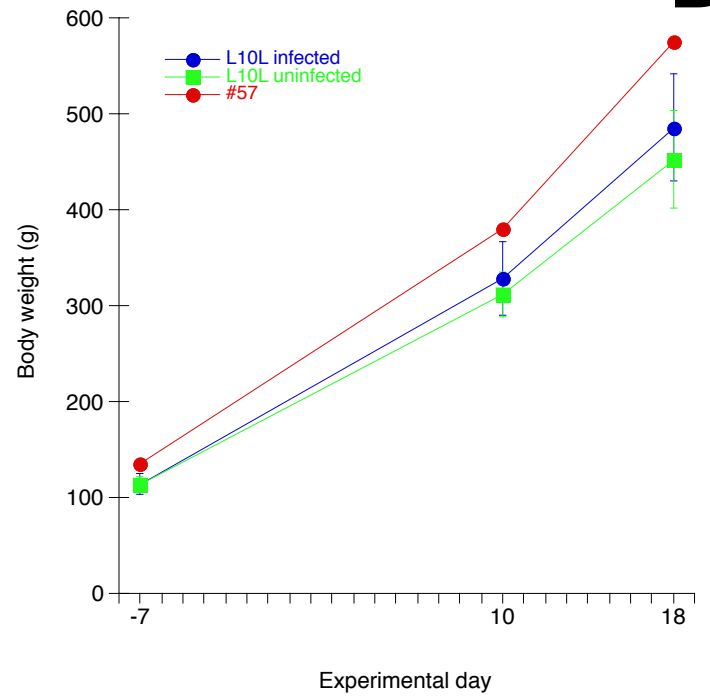**C**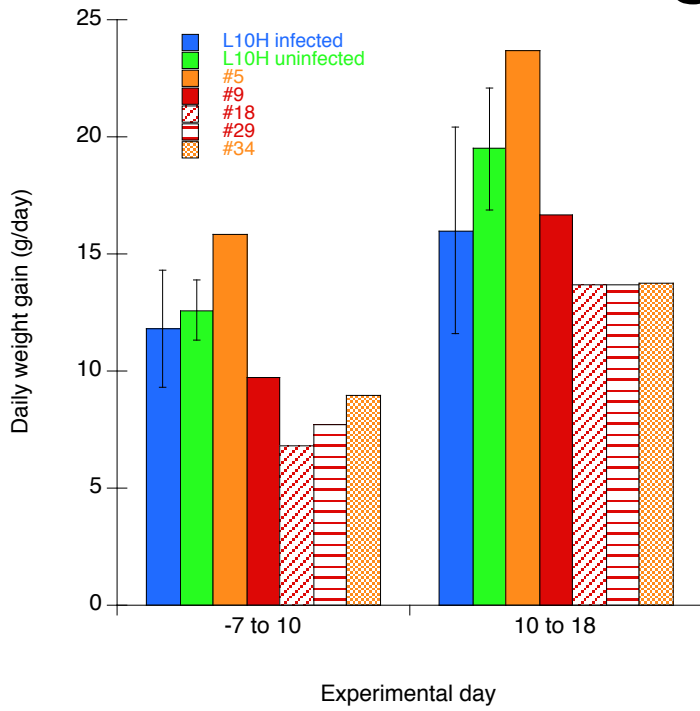**D**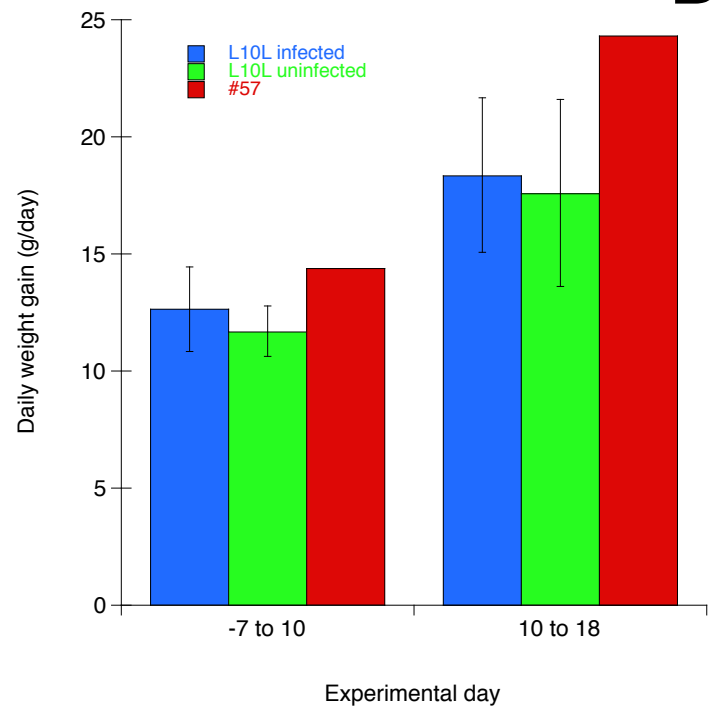

Supplement: Supplementary file 5 — Additional file 5. Body weights (A, B) and daily weight gains (C, D). Results for L10H chickens (A, C) and L10L (B, D) chickens at the indicated experimental days. Values are group means ± 95% CI for chickens infected with ER on day 0 (dark blue symbols) and for uninfected chickens (green symbols), where non-overlapping CI indicate statistically significant differences, and for individual chickens with clinical signs of disease at one or more occasions (red symbols) and chickens positive for ER in blood at one or more occasions without clinical signs of disease (orange symbols). [file 13567_2022_1126_MOESM5_ESM.pdf]

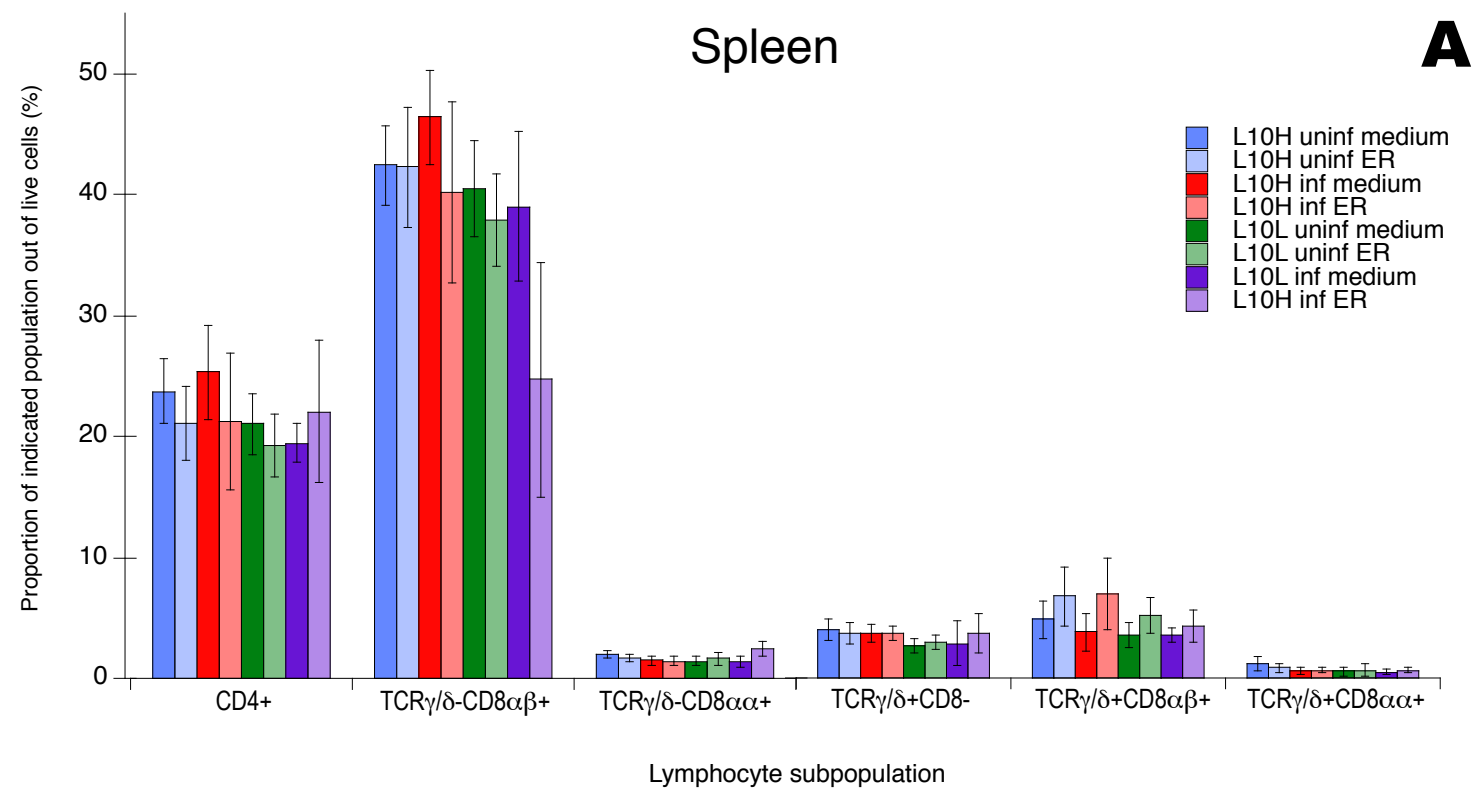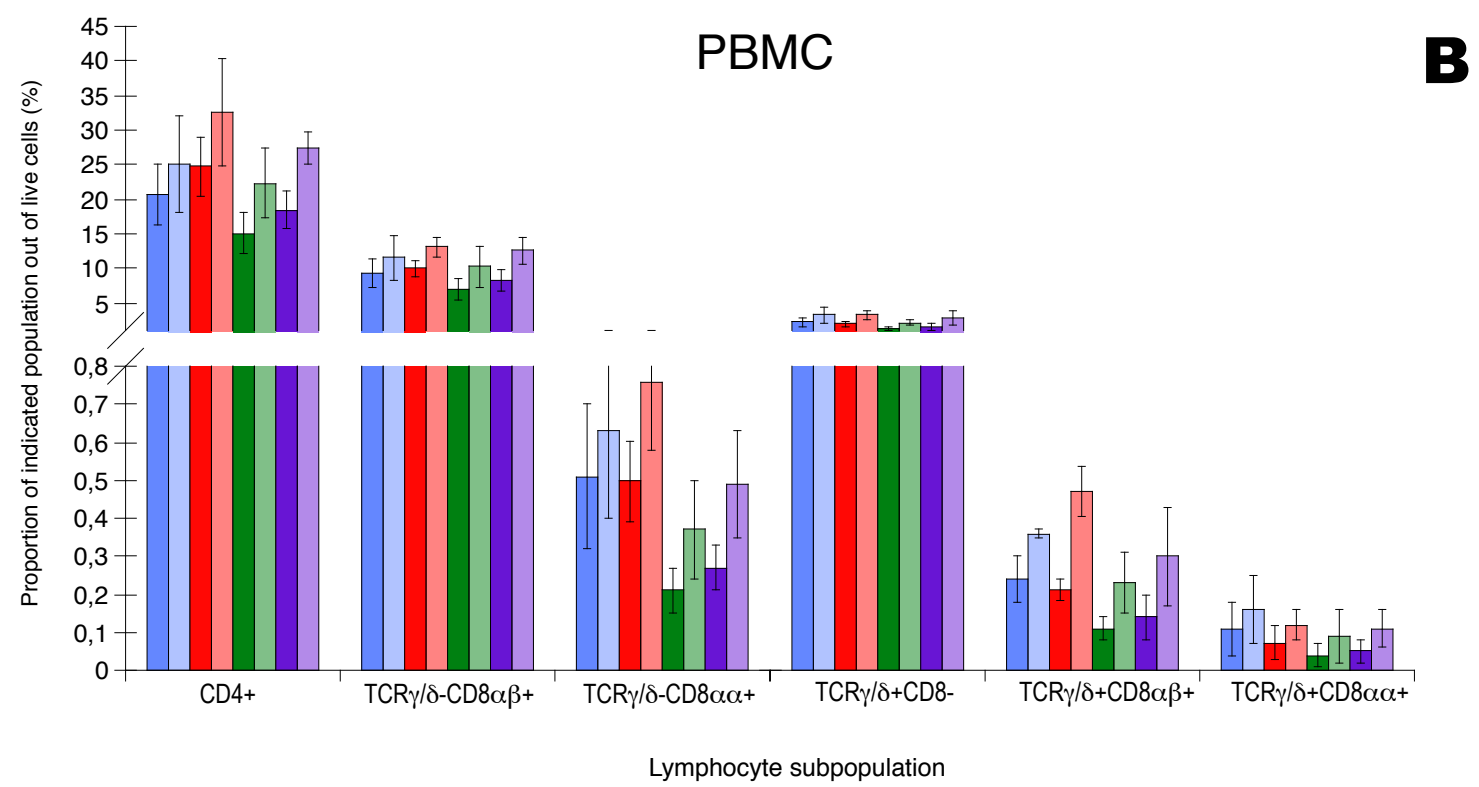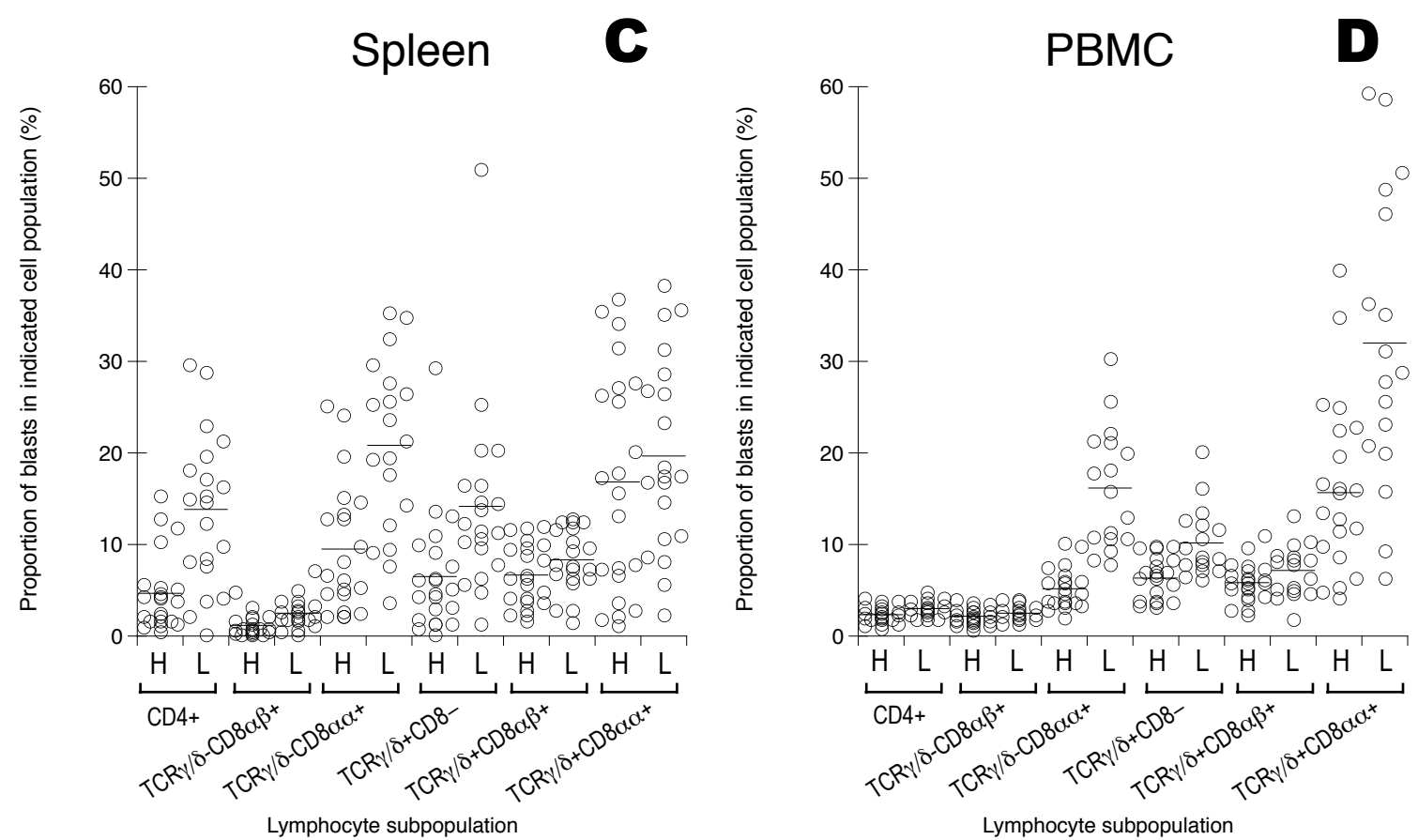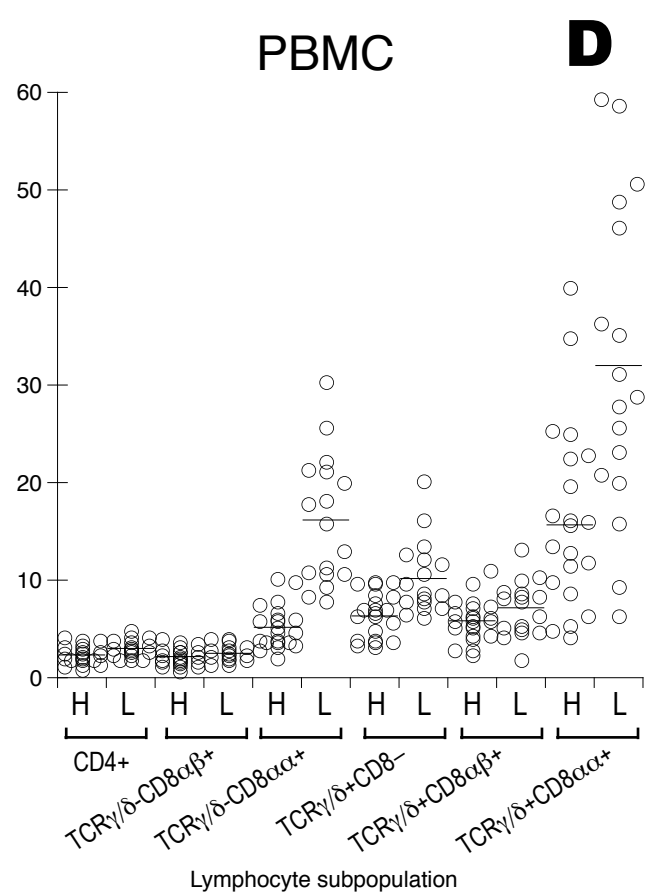

Supplement: Supplementary file 9 — Additional file 9. Proportions of the indicated lymphocyte subpopulations. Results are proportions out of live cells in spleen cells (A) or PBMC (B) from uninfected (uninf; blue bars) or ER infected (inf; red bars) L10H chickens or uninfected (uninf; green bars) or ER infected (inf; purple bars) chickens, respectively, cultured for 72 h in growth medium alone (medium; dark coloured bars) or growth medium supplemented with ER antigen (ER; light coloured bars). Spleens and blood samples were collected on day 18 after ER infection of chickens and values are group means ± 95% CI (9 ≥ n ≤ 12), where non-overlapping CI indicate statistically significant differences. Proportions of spontaneously blast transformed cells in cultures of spleen cells (C) or PBMC (D) collected on day 18 after ER infection out of the indicated lymphocyte subpopulations after 72 h of culture in growth medium alone. Individual values of all L10H (H) or L10L (L) chickens included are shown as open circles and mean values for the indicated lymphocyte subpopulations are represented by a line. Lymphocyte subpopulations were identified using monoclonal antibody panel 3 (Table 2) and the gating strategy is described in Additional file 3. [file 13567_2022_1126_MOESM9_ESM.pdf]
