# Supplementary material for: Erysipelothrix rhusiopathiae-specific T-cell responses after experimental infection of chickens selectively bred for high and low serum levels of mannose-binding lectin
Source: Vet Res. 2022 Dec 12;53:105. doi: 10.1186/s13567-022-01126-w (PMC9743643; doi:10.1186/s13567-022-01126-w)
Supplement: Supplementary file 4 — Additional file 4. Clinical signs, ER re-isolation and post-mortem findings of chickens positive for ER in blood. [file 13567_2022_1126_MOESM4_ESM.doc]

Additional file 4. Clinical signs, ER re-isolation and *post-mortem* findings of chickens positive for ER in blood after intramuscular inoculation of 0.75x10^8^ cfu ER on experimental day (Day) 0

|  |  |  |  |  | Detection of ER in blood | | |  |  |
| --- | --- | --- | --- | --- | --- | --- | --- | --- | --- |
| Group | ID | MHC | Day | Clinical signs | Culture  (cfu/ml) | Real time PCR  (copies/ml) | ddPCR  (copies/ml) | ER in liver  (culture/PCR) | *Post-mortem* |
| L10H | 2 | 193/193 | 1 | None | negative | negative |  |  |  |
|  |  |  | 2 | None | nt |  |  |  |  |
|  |  |  | 3 | Moderate; ruffled feathers, low activity but reactive when disturbed- morning  Found dead - afternoon | 1.2 x10^7^ | 1.2 x10^7^ | 5.01 x10^6^ | +/+ | Female. Splenomegaly. Pale kidneys with blood filled capillaries, uric acid crystals in tissue, and deposition of uric acid in ureters. Yellow discoloration of epicardial fat. |
|  |  |  |  |  |  |  |  |  |  |
| L10H | 5 | 193/448 | 1 | None | negative | negative |  |  |  |
|  |  |  | 2 | None | nt |  |  |  |  |
|  |  |  | 3 | None | 9.0 x10^1^ | negative |  |  |  |
|  |  |  | 4 | None | nt |  |  |  |  |
|  |  |  | 5 | None | nt |  |  |  |  |
|  |  |  | 6 | None | nt |  |  |  |  |
|  |  |  | 7 | None | nt |  |  |  |  |
|  |  |  | 8 | None | nt |  |  |  |  |
|  |  |  | 9 | None | nt |  |  |  |  |
|  |  |  | 10 | None | negative | negative |  |  |  |
|  |  |  | 14 | None | negative | negative |  |  |  |
|  |  |  | 18 | None | negative | negative |  | -/- | Male. No macroscopic lesions. |
|  |  |  |  |  |  |  |  |  |  |
| L10H | 9 | 193/448 | 1 | None | nt |  |  |  |  |
|  |  |  | 2 | None | 4.0 x10^1^ | negative | negative |  |  |
|  |  |  | 3 | None | nt |  |  |  |  |
|  |  |  | 4 | None | 1.56x10^6^ | 5.01x10^5^ | 2.25 x10^5^ |  |  |
|  |  |  | 5 | Mild; lower activity | nt |  |  |  |  |
|  |  |  | 6 | None | nt |  |  |  |  |
|  |  |  | 7 | None | 1.84 x10^4^ | 1.74 x10^5^ | 9.88 x10^4^ |  |  |
|  |  |  | 8 | None | nt |  |  |  |  |
|  |  |  | 9 | None | nt |  |  |  |  |
|  |  |  | 10 | None | 4.0 x10^1^ | negative | negative |  |  |
|  |  |  | 14 | None | negative | negative | nt |  |  |
|  |  |  | 18 | None | negative | negative | nt | -/- | Female. Blood filled capillaries in local area of the liver. |
|  |  |  |  |  |  |  |  |  |  |
| L10H | 18 | 193/193 | 1 | None | negative |  |  |  |  |
|  |  |  | 2 | None | nt |  |  |  |  |
|  |  |  | 3 | None | 2.19 x10^5^ | 2.89 x10^4^ | 1.45 x10^4^ |  |  |
|  |  |  | 4 | Mild-moderate; lower activity | nt |  |  |  |  |
|  |  |  | 5 | Moderate; low activity but reactive when disturbed | nt |  |  |  |  |
|  |  |  | 6 | Mild; lower activity | nt |  |  |  |  |
|  |  |  | 7 | Mild; lower activity | nt |  |  |  |  |
|  |  |  | 8 |  | nt |  |  |  |  |
|  |  |  | 9 |  | nt |  |  |  |  |
|  |  |  | 10 |  | 1.0 x10^1^ | 2.46 x10^2^ | negative |  |  |
|  |  |  | 14 | None | negative | negative |  |  |  |
|  |  |  | 18 | None | negative | negative |  | -/- | Female. No macroscopic lesions. |
|  |  |  |  |  |  |  |  |  |  |
| L10H | 27 | 193/193 | 1 | None | 6.5 x10^1^ | negative | negative |  |  |
|  |  |  | 2 | None | nt |  |  |  |  |
|  |  |  | 3 | Found dead - morning | nt |  |  | +/+ | Female. Splenomegaly and hepatomegaly.  Grey discoloration of kidney tissue with blood filled capillaries, uric acid crystals in tissue, and deposition of uric acid in ureters. Yellow discoloration of epicardial fat. Cloudy right thoracic air sac. |
|  |  |  |  |  |  |  |  |  |  |
| L10H | 29 | 193/193 | 1 | None | nt |  |  |  |  |
|  |  |  | 2 | None | negative | negative |  |  |  |
|  |  |  | 3 | None | nt |  |  |  |  |
|  |  |  | 4 | Moderate; low activity but reactive when disturbed, slightly hanging wings | 1.37 x10^5^ | ns | ns |  |  |
|  |  |  | 5 | Mild; lower activity | nt |  |  |  |  |
|  |  |  | 6 | Mild; lower activity | nt |  |  |  |  |
|  |  |  | 7 | None | 1.75 x10^2^ | 5.12 x10^4^ | 1.06 x10^4^ |  |  |
|  |  |  | 8 | None | nt |  |  |  |  |
|  |  |  | 9 | None | nt |  |  |  |  |
|  |  |  | 10 | None | 1.0 x10^1^ | negative | negative |  |  |
|  |  |  | 14 | None | negative | negative |  |  |  |
|  |  |  | 18 | None | negative | negative |  | -/- | Female. No macroscopic lesions. |
|  |  |  |  |  |  |  |  |  |  |
| L10H | 34 | 193/193 | 1 | None | negative | negative |  |  |  |
|  |  |  | 2 | None | nt |  |  |  |  |
|  |  |  | 3 | None | negative | negative |  |  |  |
|  |  |  | 4 | None | nt |  |  |  |  |
|  |  |  | 5 | None | nt |  |  |  |  |
|  |  |  | 6 | Very mild?; lower activity? | nt |  |  |  |  |
|  |  |  | 7 | None | nt |  |  |  |  |
|  |  |  | 8 | None | nt |  |  |  |  |
|  |  |  | 9 | None | nt |  |  |  |  |
|  |  |  | 10 | None | 6.0x10^1^ | 3.89x10^3^ |  |  |  |
|  |  |  | 14 | None | negative | negative |  |  |  |
|  |  |  | 18 | None | negative | negative |  | -/- | Female. No macroscopic lesions. |
|  |  |  |  |  |  |  |  |  |  |
| L10L | 51 | 193/193 | 1 | None | nt |  |  |  |  |
|  |  |  | 2 | None | 1.0x10^1^ | negative |  |  |  |
|  |  |  | 3 | None | nt |  |  |  |  |
|  |  |  | 4 | None apparent but died at blood sampling | 1.5x10^5^ | 2.0x10^5^ | 1.06 x10^5^ | +/+ | Male. 1.5 ml blood clot on the liver surface. |
|  |  |  |  |  |  |  |  |  |  |
| L10L | 54 | 193/193 | 1 | None | negative | negative |  |  |  |
|  |  |  | 2 | None | nt |  |  |  |  |
|  |  |  | 3 | Moderate; ruffled feathers, low activity but reactive when disturbed, died at blood sampling | 1.8x10^5^ | 2.60x10^4^ | 2.03x10^4^ | +/+ | Female. 1 ml blood clot on the liver surface. Mild splenomegaly. Kidney tissue protruding with deposition of uric acid in ureters |
|  |  |  |  |  |  |  |  |  |  |
| L10L | 57 | 193/193 | 1 | None | negative | negative |  |  |  |
|  |  |  | 2 | None | nt |  |  |  |  |
|  |  |  | 3 | None | 1.8x10^3^ | 5.25x10^2^ | negative |  |  |
|  |  |  | 4 | Mild-moderate; lower activity – in morning only | nt |  |  |  |  |
|  |  |  | 5 | None | nt |  |  |  |  |
|  |  |  | 6 | None | nt |  |  |  |  |
|  |  |  | 7 | None | nt |  |  |  |  |
|  |  |  | 8 | None | nt |  |  |  |  |
|  |  |  | 9 | None | nt |  |  |  |  |
|  |  |  | 10 | None | negative | negative |  |  |  |
|  |  |  | 14 | None | negative | negative |  |  |  |
|  |  |  | 18 | None | negative | negative |  | -/- | Male. No macroscopic lesions. |
|  |  |  |  |  |  |  |  |  |  |
| L10L | 58 | 193/193 | 1 |  |  |  |  |  |  |
|  |  |  | 2 |  |  |  |  |  |  |
|  |  |  | 3 | Moderate; ruffled feathers, low activity but reactive when disturbed – morning  Severe; standing apathetic with closed eyes, no reaction when touched – evening. Euthanized | 6.75x10^6^ | 1.07x10^6^ | 7.53x10^5^ | +/+ | Female. Hepatomegaly with small areas of hemorrhage. Some yellow discoloration of epicardial fat. Some deposition of uric acid crystals in ureters. |

nt – not tested

ns – no sample

+ - positive
