# Supplementary material for: Erysipelothrix rhusiopathiae-specific T-cell responses after experimental infection of chickens selectively bred for high and low serum levels of mannose-binding lectin
Source: Vet Res. 2022 Dec 12;53:105. doi: 10.1186/s13567-022-01126-w (PMC9743643; doi:10.1186/s13567-022-01126-w)
Supplement: Supplementary file 7 — Additional file 7. Numbers of different lymphocyte subpopulations. B-cells (A, B); TCRγ/δ+ cells (C, D); CD4+CD8− cells (E, F); CD4−CD8αβ+ cells (G, H) and CD4−CD8αα+ cells (I, J), in blood from L10H (A, C, E, G, I and K) and L10L (B, D, F, H, J and L) chickens at the indicated days after ER infection on day 0. Results are mean values ± 95% CI for infected chickens (dark blue circles) and uninfected chickens (green squares), where non-overlapping CI indicate statistically significant differences, and individual values for infected chickens. Black circles: chickens that eventually died, red circles: chickens that showed clear clinical signs of disease at one or more occasions, orange circles: chickens positive for ER in blood at one or more occasions without clear clinical signs of disease, light blue circles: infected chickens without clinical signs of disease or bacteraemia. Monoclonal antibody panels for immunolabelling are described in Table 2 and gating strategies in Additional files 1 and 2. [file 13567_2022_1126_MOESM7_ESM.pdf]

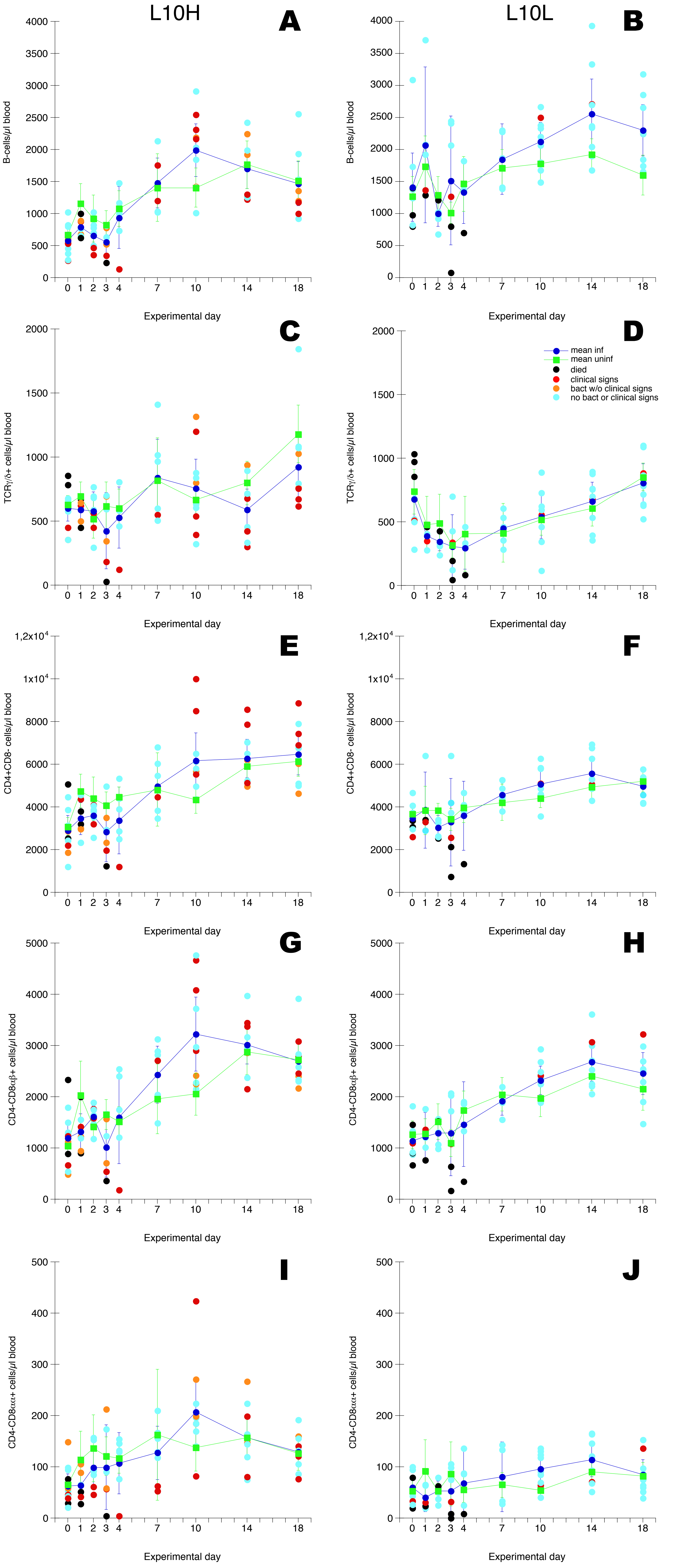

L10H
L10L
A
B
C
D
E
F
G
H
I
J
B-cells/µl blood
TCRγ/δ+ cells/µl blood
CD4+CD8- cells/µl blood
CD4-CD8αβ+ cells/µl blood
CD4-CD8αα+ cells/µl blood
Experimental day
mean inf
mean uninf
died
clinical signs
bact w/o clinical signs
no bact or clinical signs
